# Current strategies and challenges in vaccine platforms for Newcastle Disease Virus

**DOI:** 10.1186/s40794-026-00320-4

**Published:** 2026-10-30

**Authors:** Charlie Franck Amoia, Gerald Misinzo, Muhammad Munir

**Affiliations:** 1 Department of Biology-Geology, Faculty of Science and Technology, Alassane Ouattara University, Bouaké, Côte d’Ivoire; 2 SACIDS Africa Centre of Excellence for Infectious Diseases, SACIDS Foundation for One Health, Sokoine University of Agriculture, Morogoro, 67125 Tanzania; 3 OR Tambo Africa Research Chair for Viral Epidemics, SACIDS Foundation for One Health, Sokoine University of Agriculture, Morogoro, 67125 Tanzania; 4 Department of Veterinary Microbiology, Parasitology and Biotechnology, Sokoine University of Agriculture, Morogoro, 67125 Tanzania; 5 Division of Biomedical and Life Sciences, Faculty of Health and Medicine, Lancaster University, Lancaster, LA1 4YG UK

**Keywords:** Newcastle disease virus, Vaccine platforms, Antigenic diversity, Recombinant vaccines, Virus-like particles, Reverse genetics, Maternal antibody interference, DIVA, Mucosal immunity, Thermostable vaccines

## Abstract

Newcastle disease virus (NDV) remains one of the most economically devastating pathogens affecting poultry production. Despite decades of vaccination efforts, NDV continues to circulate and remain endemic across Asia, Africa, and the Middle East, posing persistent threats to food security, rural livelihoods, and international trade. This review comprehensively examines the current landscape of NDV vaccine platforms, their immunological mechanisms, and the multifaceted challenges limiting their efficacy. We critically evaluated conventional platforms, including lentogenic live attenuated vaccines and oil-adjuvanted inactivated formulations, highlighting their contributions to disease control while addressing their inherent limitations including cold-chain dependence, maternal antibody interference, incomplete cross-protection against heterologous genotypes, and the absence of differentiating infected from vaccinated animals (DIVA) capability. We then examined next-generation approaches, including recombinant vector platforms, virus-like particles, DNA and mRNA vaccines, and AI/ML-informed antigen design, assessing each against its actual stage of development, from vaccines already in commercial use to platforms that remain preclinical or theoretical, rather than treating design capability as equivalent to field-ready deployment. Finally, we addressed regulatory, manufacturing, and logistical barriers to vaccine deployment in low-resource settings, including a dedicated feasibility assessment and platform-comparison table for smallholder-dominated, endemic regions. Integrating these technological advances with robust surveillance, biosecurity, and supportive policies will be essential for achieving sustainable NDV control and advancing toward eradication.

## Introduction

### Overview of Newcastle Disease Virus

Newcastle disease virus (NDV), with taxonomic name of *Orthoavulavirus javaense*, formally classified as Avian orthoavulavirus 1 (AOaV-1), Avian paramyxovirus or Avian avulavirus 1, within the genus *Orthoavulavirus* and family *Paramyxoviridae*, stands as one of the most significant and ubiquitous threats to global avian health and food security [128]. This enveloped virus possesses a non-segmented, single-stranded, negative-sense RNA genome of approximately 15.2 to 15.6 kilobases, encoding six essential structural proteins in the order 3′-NP-P-M-F-HN-L-5′. The nucleoprotein (NP), phosphoprotein (P), and large polymerase (L) form the replication-competent ribonucleoprotein complex, while the matrix (M) protein orchestrates viral assembly [156]. The two surface glycoproteins, fusion (F) and hemagglutinin-neuraminidase (HN), are the primary mediators of host cell attachment, entry, and egress, and serve as the main targets for neutralizing antibodies. Furthermore, RNA editing of the P gene mRNA allows for the expression of two non-structural V and W proteins, which are critical virulence factors known to antagonize the host interferon response, modulating the immune landscape during infection [156].

The clinical and epidemiological significance of NDV is mainly attributed to three pathotypes, which correlate directly with the molecular determinant of virulence and the amino acid motif at the cleavage site of the F protein. *Lentogenic strains*, typically found in wild birds, possess a monobasic motif (112G/E-K/R-Q-G/E-R↓L117) cleavable by trypsin-like proteases restricted to the respiratory and intestinal tracts. In stark contrast, *mesogenic* (moderate virulent) and *velogenic* (high virulent) strains possess a polybasic motif (112R/K-R-Q-R/K-R↓F117) that is cleaved by ubiquitous furin-like proteases, enabling systemic infection and severe disease [156]. Velogenic viscerotropic strains cause devastating hemorrhagic lesions and near-total mortality, while neurotropic strains induce profound neurological signs [109]. This pathogenic potential is further complicated by substantial genetic diversity. The NDV is divided into two classes, with Class I containing a single genotype and Class II encompassing at least 21 genotypes (I-XXI). The continuous evolution and global circulation of novel genotypes, particularly the highly virulent genotype VII in Africa and Asia, pose a persistent risk of vaccine mismatch and breakdown in immunity [33].

In sub-Saharan Africa, from Côte d’Ivoire to Tanzania and Kenya, village chickens are not merely a source of food but a cornerstone of rural economies, providing essential income for women and children, serving in socio-cultural rituals, and contributing to soil fertility (C. F. A. N. G. Amoia et al., [8], Ouma, [113]. Côte d’Ivoire, for instance, backyard poultry constitutes 75% of the national flock, while in Tanzania, over 80% of households engage in poultry rearing. However, these systems are characterized by minimal biosecurity, inadequate veterinary care, and unregulated live bird markets, creating ideal conditions for the endemic persistence and spread of NDV. Outbreaks, often peaking during seasonal changes or festivals, can decimate 80–100% of a village’s flock, devastating household nutrition and financial stability [148].

Current control strategies rely heavily on vaccination, using live attenuated or inactivated vaccines. However, their field efficacy is frequently compromised. The genetic divergence between classical vaccine strains and circulating field viruses (genotype VII) can lead to incomplete protection, reducing clinical disease but failing to prevent infection and viral shedding, a phenomenon known as “leaky” immunity [34]. Furthermore, practical hurdles in low-resource settings, including the cold-chain requirement, interference from maternal antibodies, the logistical complexity of booster regimes, and the potential for reversion to virulence of live vaccines, severely limit successful implementation. Consequently, even in vaccinated populations, ND remains a potent threat, as evidenced by outbreaks in vaccinated commercial flocks in China linked to evolved vaccine strain [153].

The convergence of viral evolution, agricultural practice, and logistical limitations has catalyzed a paradigm shift towards next-generation vaccine platforms. The development of genotype-matched vaccines, particularly those based on DNA or viral vectors, represents a promising frontier. DNA vaccines, for example, offer a compelling profile as they are inherently safe with no risk of reversion, are genetically stable, can be rapidly designed to match emerging strains, and do not require a cold chain [93]. Engineering of lentogenic strains to express the F and HN proteins of a field-dominant velogenic genotypes (e.g. genotype VII) and inclusion of immunomodulators (e.g. interferon lambda-3) induce robust protection and reduce viral transmission. While these studies provide potential for field deployment, large-scale field validations are warranted in future. The strategic integration of such innovative tools with enhanced surveillance, strict biosecurity, and supportive policies is imperative to curb the enduring burden of ND, safeguard the livelihoods of millions of smallholder farmers, and ensure the sustainability of the global poultry industry in an increasingly interconnected world.

### Global impact on poultry health and industry

**Fig. 1:**
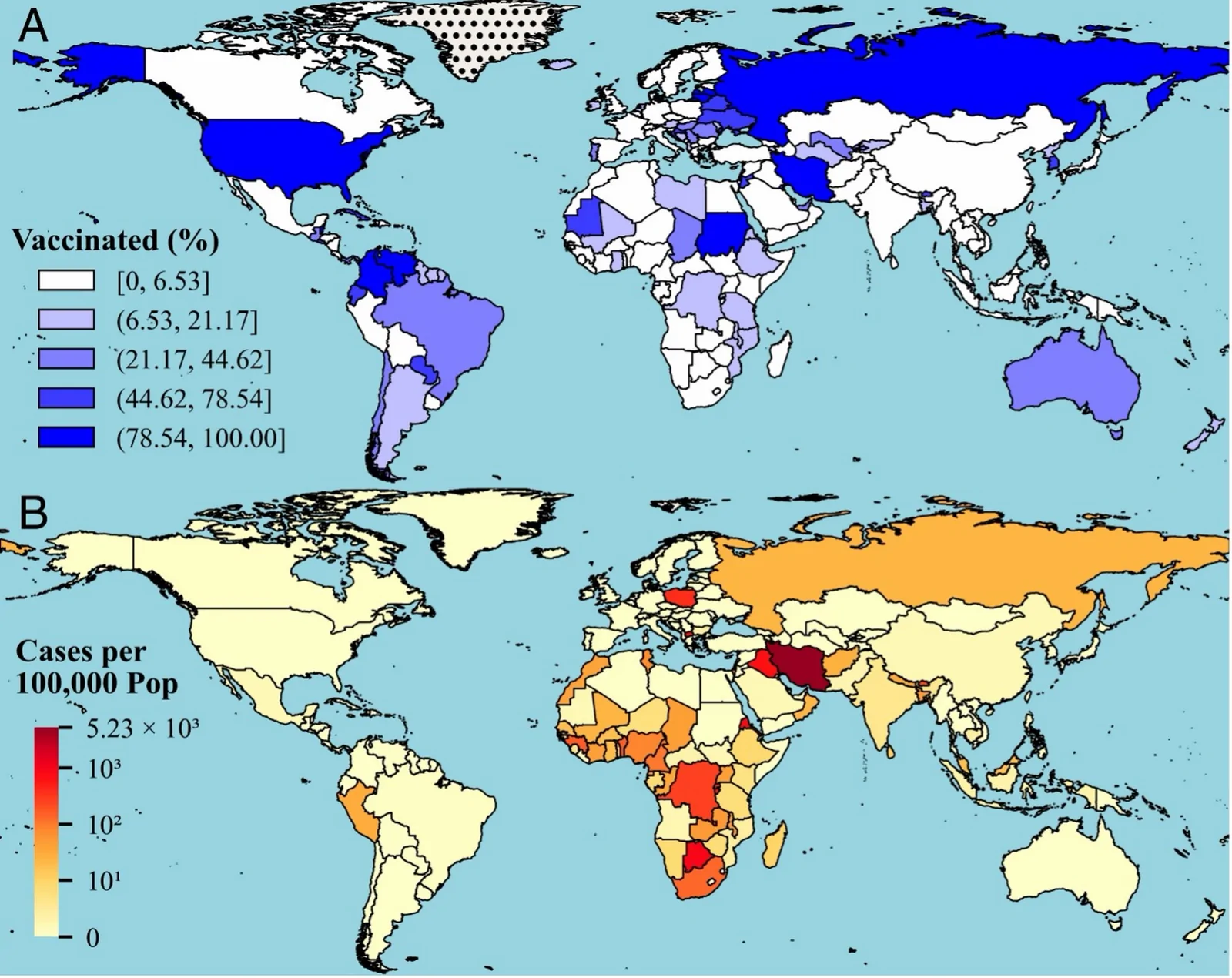
The spatial distribution of Newcastle disease and vaccination efforts in poultry during 2025. Country-level vaccination coverage is shown in (**A**), and (**B**) maps the incidence of vND per 100,000 birds. Note that cross-hatching denotes territories excluded due to a lack of reliable data

The global poultry industry faces major impacts from diseases like ND, climate change, rising feed/energy costs, and consumer demands, threatening food security, biosecurity, and profitability, leading to economic losses, trade disruptions, and environmental concerns, necessitating innovations in genetics, welfare, and sustainable practices [18]. Among the selected poultry diseases, vND exhibited the highest global incidence in 2025. Iran recorded the highest estimated number of cases at 152,614,848, despite achieving 100% vaccination coverage. This was followed by Poland (5,832,515 cases; 0.0% coverage), Iraq (2,076,904; ~0.0%), South Africa (1,527,310; 0.1%), Russia (725,581; 100.0%), and Nigeria (453,991; 0.9%) (Fig. 1) [51]. The primary economic impact stems from the catastrophic mortality associated with velogenic NDV strains, which can decimate 80–100% of an unprotected flock within days, representing a direct and total loss of capital investment [125]. Beyond acute mortality, the disease imposes severe production losses. In surviving or sub-clinically infected laying hens, NDV infection causes a dramatic and prolonged decline in egg production, often by 50% or more, alongside a significant increase in malformed, thin-shelled, or depigmented eggs, rendering them commercially non-viable [23]. In broilers, infection leads to reduced weight gain, poor feed conversion ratios, and high condemnation rates at processing due to pathological lesions, severely undermining productivity [110].

The economic ramifications extend far beyond the farm gate. The international trade in poultry and poultry products is heavily regulated based on ND status. An outbreak of velogenic ND triggers immediate international trade embargoes and the loss of “disease-free” status by the World Organisation for Animal Health (WOAH), blocking export markets and causing long-term damage to a country’s agricultural economy. The 2002–2003 outbreak of exotic ND in California, USA, required the culling of over 3.5 million birds and cost approximately $200 million in direct eradication costs and forgone trade, illustrating the scale of economic disruption in a developed economy [107].

In developing nations, where the poultry sector is often a critical component of food security and rural livelihoods, the impact is even more profound. Village chicken production, which supplies over 70% of poultry meat and eggs in many African and Asian rural communities, is persistently constrained by endemic ND [108]. Outbreaks periodically wipe out household assets, plunging families into poverty, disrupting local protein supply, and undermining nutritional security, particularly for women and children who often manage these flocks [120]. Furthermore, the costs of control measures, including emergency vaccination campaigns, surveillance, diagnostics, and enforcement of movement controls place a heavy burden on under-resourced national veterinary services [16].

The virus’s high contagiousness and environmental stability exacerbate these losses, as rapid spread within dense production areas can amplify an isolated outbreak into a regional crisis [83]. Consequently, ND is not merely a veterinary concern but a critical threat to global food security, economic stability, and the sustainable development of the rapidly growing poultry sector, especially in regions where animal protein demand is rising. This stark reality underscores the imperative for effective, accessible, and sustainable control strategies as a cornerstone of agricultural development policy [84]. Due to its high pathogenicity, ND is listed as a notifiable disease in WOAH Terrestrial Animal Health Code, requiring immediate reporting [88].

### Need for effective vaccination strategies

Vaccination has long been established as the cornerstone of ND control, a necessary intervention to mitigate the catastrophic economic and food security impacts of the virus [121].However, the static nature of many conventional vaccines is increasingly mismatched against the dynamic challenges posed by NDV epidemiology, host ecology, and global poultry production realities [67]. This discord necessitates a continuous cycle of innovation and strategic improvement in vaccination approaches.

The primary virological driver for improved vaccines is the relentless genetic and antigenic evolution of NDV. The co-circulation of multiple genotypes and the emergence of novel lineages, particularly within Class II genotypes like V, VII, and XIII, create a moving target for immunity [82]. Vaccine strains based on historical genotypes such as the globally used LaSota strain, using genotype II may provide adequate protection against mortality from heterologous challenges but often fail to prevent infection and subsequent viral shedding, a phenomenon termed “leaky” immunity [129]. This incomplete protection allows for the silent circulation of virulent field viruses within vaccinated populations, facilitating further evolution and posing a persistent threat of breakthrough outbreaks. This underscores the need for next-generation vaccines capable of eliciting broader, more sterilizing immunity, potentially through genotype-matched designs or those targeting conserved epitopes [2, 133].

Beyond virology, the vast heterogeneity of poultry production systems imposes critical practical constraints on vaccine efficacy. In intensive commercial systems, mass application methods such as *in ovo* vaccination or coarse spray in hatcheries demand vaccines with exceptional safety profiles (no post-vaccination reactions) and stability. In contrast, village poultry systems, which account for the majority of birds in developing countries, face near-insurmountable logistical hurdles: the absence of a reliable cold chain, limited access to veterinary services, the difficulty of handling and individually administering birds, and the ubiquitous presence of maternal antibodies that can neutralize live vaccines [113]. For these settings, ideal vaccines must be thermostable, administrable via easy routes like oral or eye drop, effective in the face of maternal immunity, and ultra-low cost [31, 97]. The widely used lentogenic live vaccines, while inexpensive, often fail on several of these counts, particularly regarding thermostability and susceptibility to maternal antibody interference [15].

Furthermore, the economic calculus of vaccination is paramount. A vaccine must not only be affordable to produce and purchase but must also provide a high return on investment by reliably preventing losses. Ineffective vaccination leads to “vaccination fatigue,” where producers lose confidence in and compliance with vaccination programs. Additionally, the ideal vaccine should be compatible with DIVA strategies, enabling serological surveillance to distinguish vaccine-induced antibodies from natural infection, which is crucial for outbreak investigation and proving disease freedom for trade [134].

Therefore, the development of future-proof ND vaccination strategies requires a multipronged focus. It must encompass (i) virological intelligence (surveillance to inform antigenic matching), (ii) platform innovation (exploiting viral vectors, nucleic acid technologies, and novel adjuvants for broader, stronger immunity), (iii) formulation science (developing thermostable, single-dose, and mucosal delivery systems), and (iv) socio-economic integration (designing programs that are logistically feasible and economically viable for all producers) [13, 154]. Only through such an integrated approach can vaccination truly fulfill its role as a resilient and sustainable cornerstone of global ND control.

## NDV biology and pathogenesis relevant to vaccine design

### Virus classification and genotypes

**Fig. 2:**
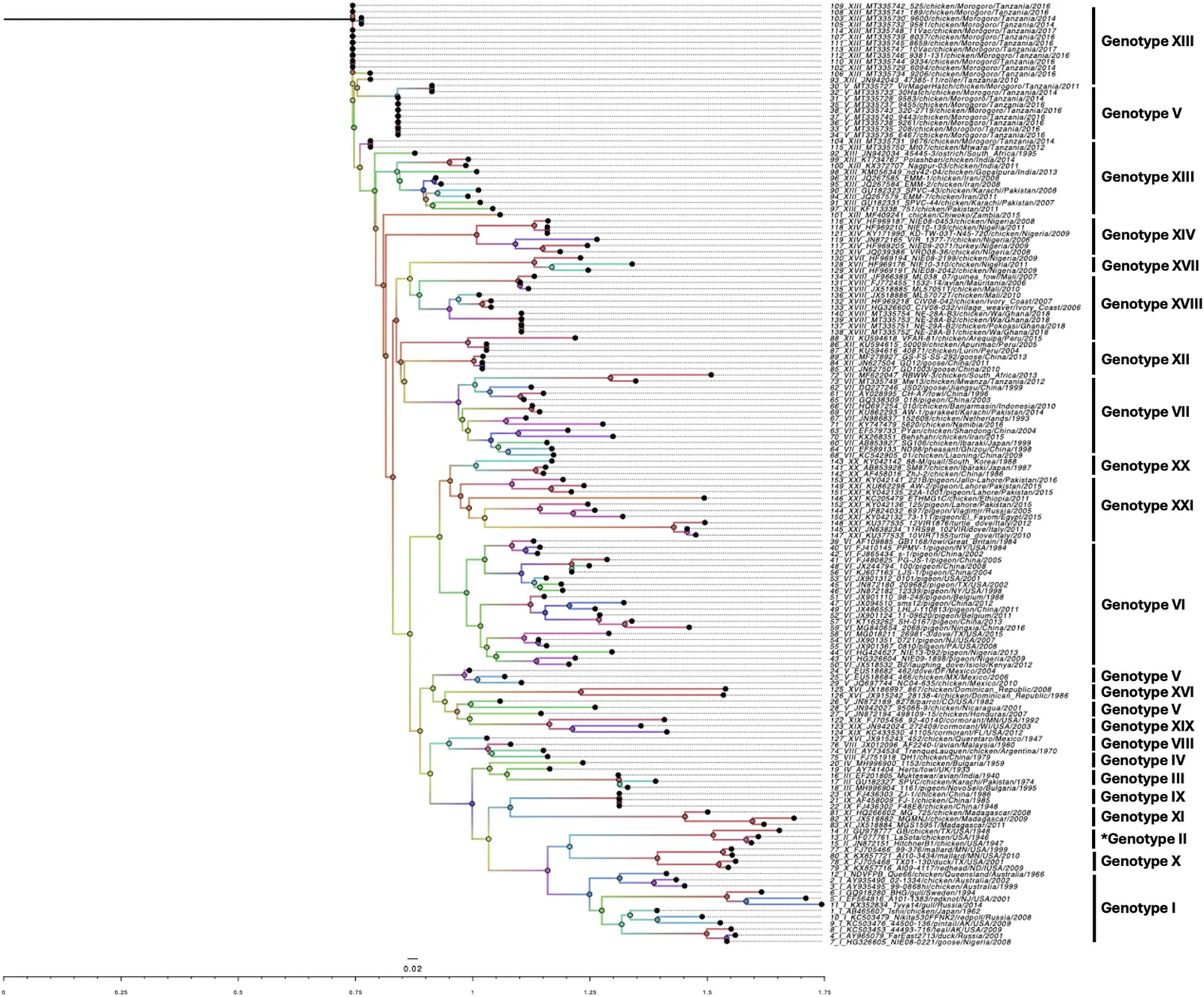
Phylogenomic analysis of representative strains of all genotypes of Class II, indicating genetic diversity of vaccine strains (marked with *) from the field strains

The genetic landscape of NDV is organized into a hierarchical classification system that is fundamental to epidemiological tracing, virulence assessment, and vaccine development. Based on phylogenetic analysis of the complete F gene sequence, NDV isolates are segregated into two distinct classes: Class I and Class II [33]. This primary division reflects deep evolutionary divergence and correlates with significant biological differences. Class I comprises a single genotype and is predominantly composed of avirulent or lentogenic viruses. These strains are frequently isolated from wild waterfowl and shorebirds, which serve as a natural reservoir, and occasionally from live bird markets. Class I viruses are genetically less diverse and are generally considered to have low pathogenic potential in chickens, though they play a crucial role in the ecology and evolution of NDV [35, 142] (Fig. 2).

In contrast, Class II is far more genetically heterogeneous and contains viruses of all pathotypes: lentogenic, mesogenic, and velogenic. This class is subdivided into at least 21 recognized genotypes, designated numerically from I to XXI [54, 103]. This extensive genotype demarcation is based on established genetic distance thresholds (typically > 10% nucleotide divergence in the F gene) and is supported by robust phylogenetic criteria. Historically, genotypes I and II include many of the classic, widely used vaccine strains. However, the epizootiological significance lies with the more recently emerged and circulating virulent genotypes. Since the late 20th century, a global panzootic has been driven primarily by virulent strains from genotypes V, VI, VII, and XIII, with specific sub-genotypes showing distinct geographical distributions [33].

Genotype V is historically linked to psittacine outbreaks and the global spread from the 1970s onward. Recent isolates in East Africa highlight its continued evolution and circulation [56]. Genotype VI is often associated with outbreaks in pigeons and doves (also referred as pigeon paramyxovirus-1, PPMV-1), with significant spillover into terrestrial poultry [59]. Genotype VII is currently the most dominant and concerning genotype in Asia, Africa, and parts of the Middle East. Sub-genotype VII.1.1 (specifically VII.1.1.1) is responsible for the majority of devastating outbreaks in vaccinated commercial and backyard flocks across these continents [82].Genotype XIII is prevalent in the Indian subcontinent and parts of Asia and Africa, contributing significantly to the ND burden in these regions [63, 78].

This profound genetic and antigenic diversity within Class II, particularly the disparity between historical vaccine genotypes (II) and contemporary outbreak genotypes (VII), is a critical consideration for vaccine design. Phylogenetic distance can correlate with antigenic divergence, potentially leading to suboptimal protection. Vaccines based on genotype II strains may prevent severe disease and mortality from a genotype VII challenge but often fail to provide sterilizing immunity, allowing for reduced clinical signs while permitting viral replication and shedding [99]. This “leaky” immunity facilitates the silent spread of virulent field viruses, complicating control and eradication efforts. Consequently, there is a strong scientific and field-driven rationale for developing and deploying genotype-matched vaccines, especially in regions where a specific virulent genotype like VII is endemic. This strategy aims to narrow the antigenic gap, improve protection, and reduce viral transmission within vaccinated populations (C. F. Amoia et al., [9, ], Hossain, [65].

### Molecular determinants of virulence

In contrast, the pathogenicity between NDV strains is fundamentally rooted in specific molecular signatures, most critically within the F protein cleavage site. The F protein is synthesized as an inactive precursor (F0) that requires proteolytic cleavage into F1 and F2 subunits to become fusion-competent and enable viral entry into host cells. The amino acid sequence at this cleavage site acts as the primary molecular determinant of tissue tropism and systemic virulence [62, 75]. Velogenic NDV strains possess a polybasic amino acid motif at residues 112–117 of the F protein (112R-R-Q-K-R-F117 or 112R-R-R-K-R-F117). This motif conforms to the recognition sequence for ubiquitous furin-like proteases present in the *trans*-Golgi network of most eukaryotic cells. Consequently, the F protein of velogenic strains is cleaved intracellularly during its transport to the cell surface, producing a fusogenic, infectious virion capable of initiating multi-cycle replication in a wide variety of tissues. This allows for systemic dissemination beyond the respiratory and enteric tracts, leading to the severe hemorrhagic, neurological, and visceral lesions characteristic of virulent ND [62, 135].

In contrast, lentogenic (low virulence) strains possess a monobasic cleavage site (^112^G-R-Q-G-R-L^117^ or ^112^G-E-Q-G-R-L^117^). This sequence is only cleaved extracellularly by trypsin-like proteases, such as secreted trypsin or specific airway proteases like TMPRSS2, which are restricted to the respiratory and intestinal tracts of birds. This cleavage dependency confines productive replication of lentogenic viruses primarily to mucosal surfaces, preventing widespread systemic infection and resulting in mild or subclinical disease [46, 115].

Beyond this canonical motif, cooperating molecular factors significantly modulate virulence. The HN protein is a critical co-determinant. Its role extends beyond receptor binding (sialic acid) and neuraminidase activity; specific mutations in the HN protein can enhance fusogenic activity in concert with the F protein, influence tissue tropism, and modulate the host immune response. For example, mutations in the HN globular head or stalk regions can alter receptor avidity and the efficiency of F protein triggering, thereby affecting the kinetics of infection and pathogenesis, even in viruses with an identical F cleavage site [76, 91]. Furthermore, other viral proteins contribute to virulence. The non-structural V protein is a potent antagonist of the host interferon (IFN) system, and variations in its sequence can affect the virus’s ability to evade the innate immune response, a key factor in establishing systemic infection [32, 118]. Recent studies also implicate the polymerase (L) protein in modulating replication fidelity and efficiency, which can influence viral load and disease progression [159]. Thus, while the F protein cleavage site is the indispensable virulence “switch,” the overall pathogenic outcome is a complex phenotype orchestrated by the interplay of multiple viral genes, each fine-tuning the virus’s ability to replicate, spread, and counteract host defenses [131].

### Host immune response to NDV infection

#### Innate immune response: The first alarm

The host immune response to NDV is a sophisticated, multi-layered defense system involving coordinated innate, humoral, and cell-mediated arms. The outcome of infection, ranging from sterilizing immunity to severe systemic disease, is dictated by the efficacy and timing of this integrated response [42, 77]. The initial defense is mounted by the innate immune system upon recognition of pathogen-associated molecular patterns (PAMPs) by host pattern recognition receptors (PRRs). NDV RNA is primarily sensed by retinoic acid-inducible gene I (RIG-I) (except for chicken where it is lacking) and melanoma differentiation-associated protein 5 (MDA5) in the cytoplasm, triggering signaling cascades that lead to the production of type I interferons (IFN-α/β) and pro-inflammatory cytokines [150, 156].This rapid IFN response establishes an antiviral state in neighboring cells by upregulating interferon-stimulated genes (ISGs), effectively limiting early viral replication. However, NDV, particularly virulent strains, employs countermeasures, primarily through their V proteins, which act as a potent IFN antagonist by blocking MDA5 signaling and STAT protein activation, thereby delaying and dampening this critical early alarm [29].

#### Humoral immunity: The neutralizing shield

The humoral response is a primary correlate of protection and is critical for preventing initial infection and viremic spread. Neutralizing antibodies, predominantly targeting the surface glycoproteins HN and F, are elicited within 7–10 days post-infection or vaccination [94]. Anti-HN antibodies block viral attachment to sialic acid receptors on host cells, while anti-F antibodies can prevent the conformational changes required for membrane fusion [141]. These antibodies are measured via the hemagglutination inhibition (HI) assay, and titers ≥ 4 log₂ (1:16) are considered protective against clinical disease. While systemic IgG provides long-term systemic protection, mucosal IgA at the portals of entry, the respiratory and digestive tracts is crucial as a first-line defense. Secretory IgA (sIgA) can neutralize the virus at mucosal surfaces, limiting initial replication and reducing shedding, a key factor in controlling transmission [106, 119].

#### Cell-mediated immunity: The cellular clean-up crew

Cell-mediated immunity (CMI) is indispensable for clearing established infection and establishing immunological memory. CD8 + cytotoxic T lymphocytes (CTLs) recognize viral peptides presented by MHC class I molecules on infected cells and directly lyse them, terminating viral production. Concurrently, CD4 + T helper (Th) cells, particularly the Th1 subset, are essential for orchestrating the adaptive response where they provide help for B cell antibody class-switching and affinity maturation and secrete cytokines like IFN-γ that enhance macrophage activity and CTL proliferation [77]. A robust CMI response is vital for protection against antigenically variant strains, as T cells often recognize more conserved internal viral epitopes (from NP, M proteins) compared to the surface-glycoprotein-focused antibody response. This provides a degree of heterologous protection and contributes to long-term immunity, even when neutralizing antibody titers wane [127].

#### Interplay and correlates of protection

Ultimate protection against NDV is not mediated by a single arm but by their synergistic interplay. Mucosal IgA and innate defenses provide immediate containment. High titers of systemic neutralizing antibodies are the best-established correlate for preventing mortality and severe disease. However, sterilizing immunity complete prevention of infection and shedding, requires the addition of a robust, rapid CMI response to eliminate infected cells before they produce significant progeny virus. Vaccine strategies that effectively stimulate this triad (mucosal IgA, systemic neutralizing antibodies, and potent CMI) are therefore most likely to confer robust and durable protection, including against heterologous viral challenges [104].

## Conventional NDV vaccine platforms

### Live attenuated vaccines

#### Lentogenic and mesogenic strains

Live attenuated vaccines (LAVs) constitute the backbone of global NDV control, leveraging the ability of replication-competent, attenuated viruses to induce broad and robust immunity. These vaccines are categorized based on the pathogenicity of the parent strain. Lentogenic strains are the most widely employed globally. The LaSota strain is the global workhorse, prized for its strong immunogenicity, which stems from robust replication in mucosal tissues, effectively stimulating both humoral and cell-mediated responses [34]. Other lentogenic strains include B1 and B1/47, V4, and the I-2 strain, which was specifically selected for enhanced heat resistance for use in village poultry systems without a cold chain [11, 94]. Mesogenic strains, such as Mukteswar and Komarov, were historically used for booster vaccinations in older birds due to their stronger antigenic stimulus. However, their use is now restricted or banned in many countries due to safety concerns, as they can cause significant morbidity, reduced egg production, and have a higher potential for reversion to virulence or spread to susceptible birds [65].

The mechanism of attenuation in these vaccines is primarily natural or empirically selected. For lentogenic vaccines, attenuation is inherent to their low-pathogenicity phenotype, characterized by the monobasic F protein cleavage site. They are administered via mass application routes: ocular, nasal (spray), or oral (drinking water), which mimic natural infection, thereby effectively stimulating local mucosal IgA production, a critical first line of defense in the respiratory tract [94].

#### Efficacy and limitations

Live attenuated NDV vaccines are highly effective due to their ability to mimic a natural infection, eliciting a comprehensive and robust immune response. Their efficacy is characterized by the induction of strong humoral immunity, including systemic IgG and, critically, secretory IgA at mucosal surfaces of the respiratory and digestive tracts, which serves as a first line of defense against viral entry [71]. Furthermore, they stimulate potent cell-mediated immunity (CMI), including CD4 + helper and CD8 + cytotoxic T lymphocytes, which are essential for clearing virus-infected cells and establishing durable immunological memory [77]. This multifaceted response provides solid protection against clinical disease and mortality from homologous and, to a significant degree, heterologous virulent challenges [133]. However, this efficacy is counterbalanced by several significant limitations that constrain their use and drive the need for alternative platforms. Replication of live vaccines, particularly the more immunogenic lentogenic strains such as LaSota, can cause mild to moderate respiratory signs. In young birds or flocks with concurrent infections, these reactions can escalate into severe air sacculitis, leading to increased mortality, reduced weight gain in broilers, and drops in egg production in layers, a direct economic cost [144]. The presence of MDA, transferred from vaccinated hens to their progeny via the yolk, is a major cause of vaccination failure in young chicks. These circulating antibodies can neutralize the live vaccine virus before it can establish a productive infection and stimulate an active immune response. This creates a critical “window of susceptibility” between the waning of protective MDA and the successful “take” of the vaccine, during which chicks are vulnerable to field virus infection [15]. Because well-characterized lentogenic strains have a stable genetic basis for attenuation, this risk is considered very low; however, it is not zero. Genetic drift or recombination in the field could theoretically restore virulence. The risk is historically higher for mesogenic strains, and evidence suggests that extensive use of certain live vaccines has contributed to the evolution of new virulent lineages in some regions [26]. Conventional LAVs induce antibodies against all major viral proteins, making it impossible to distinguish serologically between a vaccinated bird and one that has been naturally infected with a field virus. This absence of DIVA strategy severely hampers disease surveillance, outbreak investigation, and efforts to prove disease-free status for trade [95]. Most commercial LAVs are lyophilized but still require a consistent cold chain (2–8 °C) for storage and transport to maintain potency. This requirement poses a formidable logistical and economic challenge in remote, resource-limited, or tropical settings where village poultry farming is predominant, often leading to vaccine failures due to inactivation [122]. These limitations underscore why live attenuated vaccines, while indispensable, are often used as part of a priming regimen or in conjunction with other vaccine technologies (inactivated boosters, recombinant vectors) to create more effective and practical control programs.

### Inactivated (Killed) vaccines

#### Production methods

Inactivated NDV vaccines are produced through a standardized, multistep process designed to preserve antigenicity while eliminating infectivity. The first stage involves the propagation of a high-titer seed virus, typically a lentogenic strain like LaSota for safety and ease of handling, or occasionally a velogenic strain to potentially broaden antigenic coverage. This propagation occurs in the allantoic cavity of SPF embryonated chicken eggs. Following harvest and clarification, the viral suspension undergoes chemical inactivation using agents such as binary ethylenimine (BEI) or beta-propiolactone (BPL). These compounds alkylate viral nucleic acids, rendering the virus replication-incompetent while causing minimal denaturation to the essential surface glycoproteins (F and HN) [60, 79]. The final and critical step is formulation, where the inactivated antigen is homogenized with a water-in-oil emulsion adjuvant. Mineral oil-based adjuvants, often combined with emulsifiers, create a depot at the injection site, facilitating the slow release of antigen and significantly enhancing the magnitude and duration of the humoral immune response [3].

#### Immunogenicity and safety considerations

Inactivated (killed) NDV vaccines present a distinct immunological profile defined by their safety and specific immune induction characteristics, making them a crucial component of structured vaccination programs, particularly for long-lived poultry. The immunogenic strength of inactivated vaccines is almost exclusively directed toward the humoral arm of the immune system. When emulsified in potent oil-based adjuvants (typically water-in-oil), they induce high, persistent, and uniform titers of systemic neutralizing antibodies (IgG). This provides robust and long-lasting protection against viremia and clinical disease, making them excellent for securing long-term immunity in layers and breeders [6, 7].

However, this comes with significant immunological gaps. As non-replicating antigens, they poorly stimulate cytotoxic CD8 + T lymphocytes, which are critical for clearing virus-infected cells. They fail to elicit secretory IgA (sIgA) at respiratory and intestinal mucosal surfaces. Consequently, vaccinated birds are not protected against initial infection and viral replication at the portal of entry, allowing for potential viral shedding even if systemic disease is prevented [19]. They are ineffective as standalone primary vaccines. Their use mandates a prime-boost regimen: a live attenuated vaccine (administered via ocular, spray, or water) is first used to “prime” the immune system by establishing mucosal immunity and CMI. The inactivated vaccine is then administered as a booster via individual intramuscular or subcutaneous injection to elicit the high, durable antibody titers required for long-term protection [1]. Their foremost advantage is exceptional safety. With the viral genome irreversibly inactivated, there is zero risk of replication, reversion to virulence, horizontal spread, or induction of vaccine-associated disease. This makes them safe for use in birds of any age and health status. Unlike live vaccines, the inactivated viral antigen is not neutralized by circulating MDA at the injection site. This allows for effective vaccination of young chicks with persistent MDA, provided the priming live vaccine has been successfully administered after MDA waned [123]. The requirement for individual bird handling and injection is labor-intensive, stressful for the birds, and increases the risk of injury and bacterial contamination. The oil adjuvant can cause localized granulomatous reactions. Furthermore, vaccine efficacy is highly dependent on proper injection technique and depot formation; shallow or misplaced injections can lead to rapid antigen clearance and a suboptimal immune response, resulting in uneven protection within a flock [10].

## Advanced and emerging NDV vaccine technologies

### Recombinant vector vaccines

#### NDV-vectored vaccines for poultry

Recombinant vector vaccines utilize genetically modified, replication-competent viruses as vehicles to deliver and express immunogenic antigens from a target pathogen. This platform capitalizes on the vector’s ability to stimulate robust innate and adaptive immune responses while presenting the foreign antigen in its native conformation [145]. NDV itself has emerged as a highly promising viral vector for poultry vaccines. Its advantages are manifold including natural avian pathogen with a well-understood biology, possesses inherent adjuvant properties due to its ability to stimulate interferon responses, and its genome can accommodate foreign genetic inserts without significant loss of replication fitness [30]. A major focus of NDV-vectored vaccine development has been the construction of recombinant NDV expressing the H5 hemagglutinin gene of avian influenza virus (AIV), designated rNDV-H5. Numerous experimental studies have demonstrated that these constructs confer robust bivalent protection against both velogenic NDV and highly pathogenic avian influenza (HPAI) H5Nx subtypes ([72]; D. H. Kim et al., [80].The rNDV-H5 vaccine is typically administered via the oculo-nasal route or intramuscular injection, inducing strong mucosal, humoral, and cellular immune responses against both pathogens. Experimental challenge studies have shown that vaccinated birds are protected from clinical disease and mortality following lethal doses of either virus, while significantly reducing viral shedding [72]. The platform’s flexibility allows for rapid updating of the H5 insert to match emerging antigenic variants of AIV, a critical advantage over traditional inactivated influenza vaccines. Furthermore, the inherent thermostability of certain NDV vectors can be leveraged to develop thermostable rNDV-H5 formulations suitable for use in resource-limited settings where cold-chain infrastructure is unreliable [86].

The development of rNDV vectors is facilitated by reverse genetics systems, allowing precise insertion of foreign genes (from infectious bronchitis virus (IBV), infectious bursal disease virus (IBDV), or avian influenza (AIV) into non-essential intergenic regions of the NDV genome, often between the P and M or HN and L genes. These recombinant constructs replicate like the parental NDV strain, expressing the foreign protein on infected cell surfaces, thereby eliciting a targeted immune response. A key safety feature is that the NDV vector backbone is typically a well-characterized, lentogenic strain, ensuring an excellent safety profile while maintaining high immunogenicity [130].

#### Heterologous antigen expression

The versatility of recombinant NDV as a vector extends far beyond avian influenza, enabling the development of bivalent or multivalent vaccines against a spectrum of economically critical poultry pathogens. By engineering the NDV genome to express key immunogenic proteins from other viruses, a single vaccine construct can induce simultaneous protection against multiple diseases, streamlining vaccination schedules and reducing production costs. The strategy involves the insertion of foreign gene cassettes into non-coding or intergenic regions of the NDV genome using reverse genetics, ensuring the stable expression of the heterologous antigen without crippling viral replication. The expressed antigens are presented in their native conformation, eliciting robust and pathogen-specific immune responses. Key examples demonstrate this platform’s potential. rNDV vectors expressing the VP2 protein of IBDV have been successfully developed. VP2 is the major host-protective antigen responsible for inducing neutralizing antibodies. Chickens vaccinated with such rNDV-VP2 constructs developed significant anti-IBDV antibodies and were protected against virulent IBDV challenge, while also being protected against NDV [27, 90]. Given the high genetic variability of IBV, rNDV vectors expressing the S1 spike glycoprotein, which contains virus-neutralizing epitopes and serotype-determining regions, offer a flexible approach. Vaccination with rNDV-S1 can induce IBV-specific neutralizing antibodies and reduce tracheal viral loads post-challenge, providing cross-protective potential against multiple IBV serotypes when combined with conserved antigen designs [144]. While traditional live Marek’s disease virus (MDV) vaccines are already effective vectors, rNDV expressing MDV glycoproteins like gB presents an alternative strategy. This approach aims to combine the potent cell-mediated immunity induced by NDV with protection against MDV-induced lymphomas [61]. Research has explored rNDV expressing antigens from avian metapneumovirus (aMPV), fowlpox virus (FPV), and salmonella, highlighting the platform’s broad applicability [123]. A significant advantage of the NDV vector is its inherent ability to stimulate strong mucosal, humoral, and cell-mediated immunity. When administered via mucosal routes, these recombinant vaccines can induce local IgA at the respiratory tract, a critical advantage for pathogens like IBV and aMPV. Furthermore, they can be designed with DIVA capabilities by expressing only a subset of the target pathogen’s proteins, allowing serological distinction between vaccinated and field-virus-infected birds [52]. This platform represents a pivotal step towards rational, multivalent vaccine design for integrated poultry disease control.

### Viral-like particle-based vaccines

Virus-like particles (VLPs) represent a sophisticated and highly promising platform within the next generation of NDV vaccines. VLPs are non-infectious, self-assembling nanostructures that mimic the native conformation and repetitive antigenic display of a live virus but contain no viral genetic material. This design combines the exceptional safety profile of a subunit vaccine, with absolutely no risk of replication, reversion, or recombination, with the strong immunogenicity typically associated with live attenuated viruses [121]. For NDV, VLPs are generated by co-expressing the key structural proteins, primarily the matrix (M) protein, fusion (F) protein, and hemagglutinin-neuraminidase (HN) protein in heterologous expression systems such as insect cells (using baculovirus vectors), mammalian cells, or even plants. The M protein drives the budding and assembly of the particle, while the F and HN glycoproteins are incorporated into the lipid envelope, presenting their critical neutralizing epitopes in an authentic, three-dimensional context [139, 143].

The immunogenic superiority of VLP-based NDV vaccines stems from several factors. The high-density, ordered array of F and HN proteins on the VLP surface efficiently cross-links B cell receptors, leading to strong B cell activation, potent neutralizing antibody responses, and long-lived memory B cells without the need for potent adjuvants [152]. Proteins presented on VLPs often assume a native, pre-fusion conformation, which is essential for eliciting antibodies that effectively block viral entry. This is a significant advantage over some recombinant subunit vaccines where proteins may misfold. The particulate nature and size (20–200 nm) of VLPs make them ideal for uptake by antigen-presenting cells (APCs). They can also stimulate innate immune pathways through pathogen-associated molecular patterns (PAMPs), promoting a robust adaptive response [70].

Experimental studies have consistently demonstrated that VLPs containing NDV F and HN proteins induce high titers of virus-neutralizing antibodies and confer solid protection against lethal NDV challenge in chickens, often matching or exceeding the protection offered by inactivated vaccines [143]. Furthermore, the platform is inherently modular and flexible. It allows for the incorporation of antigens from other pathogens (creating multivalent VLPs) or the addition of molecular adjuvants. The production process is also scalable in controlled bioreactor systems, avoiding the reliance on embryonated eggs. Despite these advantages, challenges remain in cost-effective, large-scale manufacturing and ensuring long-term stability of the complex protein structures. However, as production platforms mature, VLP-based vaccines hold immense potential for developing safe, effective, and potentially thermostable next-generation vaccines against NDV.

Several practical constraints currently limit this potential to high-income, integrated production contexts rather than the low-resource endemic settings that are the priority target of this review. Current NDV VLP manufacturing relies almost exclusively on insect-cell/baculovirus or plant-based expression systems, both of which require specialized bioreactor facilities, controlled purification suites, and technical expertise that are concentrated in a small number of high-income countries; establishing this capability in endemic regions would require substantial capital investment and technology transfer rather than incremental scale-up of existing infrastructure. Reflecting this manufacturing complexity, VLP production is consistently reported to be substantially more expensive per dose than conventional live or inactivated vaccines, although independently verified, NDV-specific cost comparisons are limited and should be treated with appropriate caution pending further data. Critically, no NDV VLP vaccine has yet received commercial regulatory approval anywhere, and the anticipated regulatory pathway and timeline for such approval have not been clearly defined. Additionally, published protection data are correspondingly derived from experimental challenge studies rather than large-scale commercial field trials, and claims of protective efficacy should be read with this limitation in mind. Taken together, VLP platforms are, for the foreseeable future, best positioned to serve high-income, vertically integrated poultry operations, whereas optimized conventional vaccines are likely to remain the more practical option for low-resource endemic regions until manufacturing and regulatory barriers are substantively addressed.

### Nucleic acid (DNA and mRNA) vaccines

Nucleic acid vaccines represent a paradigm shift in vaccinology, offering a highly flexible, rapid, and precise platform. By delivering genetic blueprints (DNA or mRNA) encoding viral antigens directly into host cells, they harness the cell’s own machinery to produce the target immunogens, thereby mimicking aspects of a natural viral infection without the risks associated with live pathogens. Both DNA and mRNA platforms offer the potential for multivalent vaccine development through the co-delivery of multiple antigen-encoding sequences. Their chemical synthesis allows for precise quality control and eliminates the risk of contaminating adventitious agents [82].

These properties position nucleic acid platforms as promising longer-term candidates for next-generation NDV control, particularly against antigenically drifting genotypes - a promise that remains contingent on substantially reducing production costs and developing delivery systems suited to poultry. As detailed in Sections “DNA vaccines” and “mRNA vaccines” below, both DNA and mRNA NDV vaccines are presently at the preclinical stage, with no licensed product, and their translation into field-ready tools should be regarded as a longer-term goal rather than an imminent outcome.

#### DNA vaccines

DNA vaccines against NDV typically consist of a bacterial plasmid engineered to express the F and/or HN glycoprotein under the control of a strong eukaryotic promoter. Upon intramuscular or *in ovo* injection, the plasmid is taken up by host cells, leading to endogenous antigen synthesis and presentation via both MHC class I and II pathways [151]. This process effectively stimulates a balanced immune response, including production of neutralizing antibodies against the expressed glycoproteins (Humoral immunity), and induction of antigen-specific CD8 + cytotoxic T lymphocytes (CTLs) and CD4 + T helper cells, which are crucial for clearing infected cells and providing heterologous protection (Cell-mediated immunity (CMI). While early DNA vaccines often suffered from moderate immunogenicity in large animals, strategies such as codon optimization, electroporation-enhanced delivery, and co-delivery with molecular adjuvants have significantly improved their efficacy [124]. Their advantages include thermostability, ease of large-scale manufacturing, and inherent safety with no risk of reversion. These advantages must, however, be weighed against substantial practical constraints that temper near-term deployment expectations. Plasmid DNA is intrinsically a weak immunogen in avian species, and most NDV DNA vaccine candidates achieve protective immunogenicity only with electroporation or needle-free jet-injection delivery; both approaches add specialized equipment, trained operators, and per-bird handling time that meaningfully increase cost and logistical complexity relative to conventional vaccination, particularly for smallholder systems. Reported per-dose costs for electroporation-delivered or otherwise adjuvanted DNA platforms are generally estimated to be at least an order of magnitude higher than those of conventional live or inactivated vaccines, although precise, independently verified cost figures specific to NDV DNA vaccines remain scarce in the published literature and should be treated as indicative rather than definitive. The overall development pathway is also considerably longer than the speed of plasmid design might suggest - progression from construct design to manufacturing scale-up, quality control, regulatory dossier preparation, and field efficacy validation for a novel platform typically spans several years, even before in-country registration in additional jurisdictions is considered. At present, NDV DNA vaccines remain at the preclinical to early experimental stage, with no product licensed for commercial use. Critically, a realistic deployment horizon for low-resource endemic settings is on the order of 5–10 years, contingent on parallel investment in delivery-device infrastructure and regulatory capacity.

#### mRNA vaccines

The mRNA vaccine technology has been propelled to the forefront by its success in human medicine and is now rapidly advancing in veterinary applications. For NDV, lipid nanoparticle (LNP)-encapsulated mRNA vaccines encoding the F or HN proteins have shown remarkable promise in preclinical studies [48]. The LNP protects the fragile mRNA from degradation, facilitates cellular entry (often via endocytosis), and possesses inherent adjuvant properties. Once inside the cytoplasm, the mRNA is directly translated into the target antigen, leading to high levels of protein expression. Recent studies demonstrate that a single dose of an LNP-formulated mRNA vaccine encoding the NDV F protein can induce rapid, high-titer neutralizing antibodies and potent T cell responses, conferring complete protection against lethal homologous challenge in chickens [45]. The platform’s key strengths are its exceptional speed and adaptability. The vaccine sequence can be rapidly designed and synthesized once the genetic sequence of a new circulating strain is known, enabling a swift response to emerging variants. Production is also highly scalable in cell-free systems, avoiding egg-based or cell-culture limitations.

### Subunit and peptide-based vaccines

Subunit and peptide-based vaccines represent the most reductionist approach in NDV vaccinology, focusing on delivering discrete, defined antigenic components rather than whole or partial viruses. This strategy maximizes safety by eliminating all non-essential viral genetic material and structural components, thereby precluding any risk of replication, reversion, or adventitious agents. Subunit vaccines typically involve the production and purification of one or more key viral proteins, most commonly the F and HN glycoproteins, which are the primary targets for neutralizing antibodies. These proteins can be produced in heterologous expression systems such as baculovirus/insect cell systems, capable of post-translational modifications that help preserve native protein conformation, or yeast, offering high-yield and cost-effective production suitable for scaling, or plant-based systems, an emerging platform for low-cost, scalable “molecular farming” [155]. Peptide-based vaccines take this a step further by using short, synthetic peptides corresponding to specific linear B-cell or T-cell epitopes identified within the F or HN proteins. This approach allows for extreme precision, targeting only the most immunodominant and conserved regions, which could theoretically enable broad protection across genotypes [25].

The principal advantage of these platforms is their exceptional safety profile. They are non-infectious and cannot cause disease. However, their major limitation is suboptimal immunogenicity. Purified proteins and short peptides often lack the complex tertiary structure and repetitive antigenic array present on whole virions or VLPs, leading to weak B-cell activation. They are also poor inducers of cytotoxic T lymphocyte (CTL) responses, as peptides require cross-presentation pathways that are less efficient than endogenous antigen synthesis triggered by live or nucleic acid vaccines [73]. Consequently, their efficacy is almost entirely dependent on advanced adjuvant and delivery technologies. Traditional aluminum-based adjuvants are often insufficient. Research focuses on potent adjuvants such as immune-stimulating complexes (ISCOMs), oil-in-water emulsions, saponins (Quil-A), and Toll-like receptor (TLR) agonists are used to enhance innate immune recognition and promote a stronger Th1/Th2 response [41]. Also, these adjuvants act as an advanced delivery system. To improve uptake and presentation, antigens are encapsulated in biodegradable nanoparticles, liposomes, or virus-like particles (VLPs). These systems protect the antigen, facilitate dendritic cell uptake, and can provide controlled release, acting as both a delivery vehicle and an adjuvant [157]. Finally, as epitope scaffolding, an innovative approach involves grafting the target peptide epitope onto a structurally stable protein scaffold. This can present the epitope in a more immunogenic, multivalent format, mimicking the repetitive display seen on viruses [81].

While currently less efficacious than whole-virus vaccines in practical settings, subunit and peptide platforms hold long-term promise for developing thermostable, chemically defined, and potentially universal vaccines. They are particularly attractive for creating DIVA-compliant vaccines, as immunization with a single protein allows for serological screening using antibodies against other proteins. Continued advances in structural vaccinology, adjuvant science, and nano-delivery are critical to unlocking their full potential for NDV control.

### Reverse genetics for rational vaccine design

**Fig. 3:**
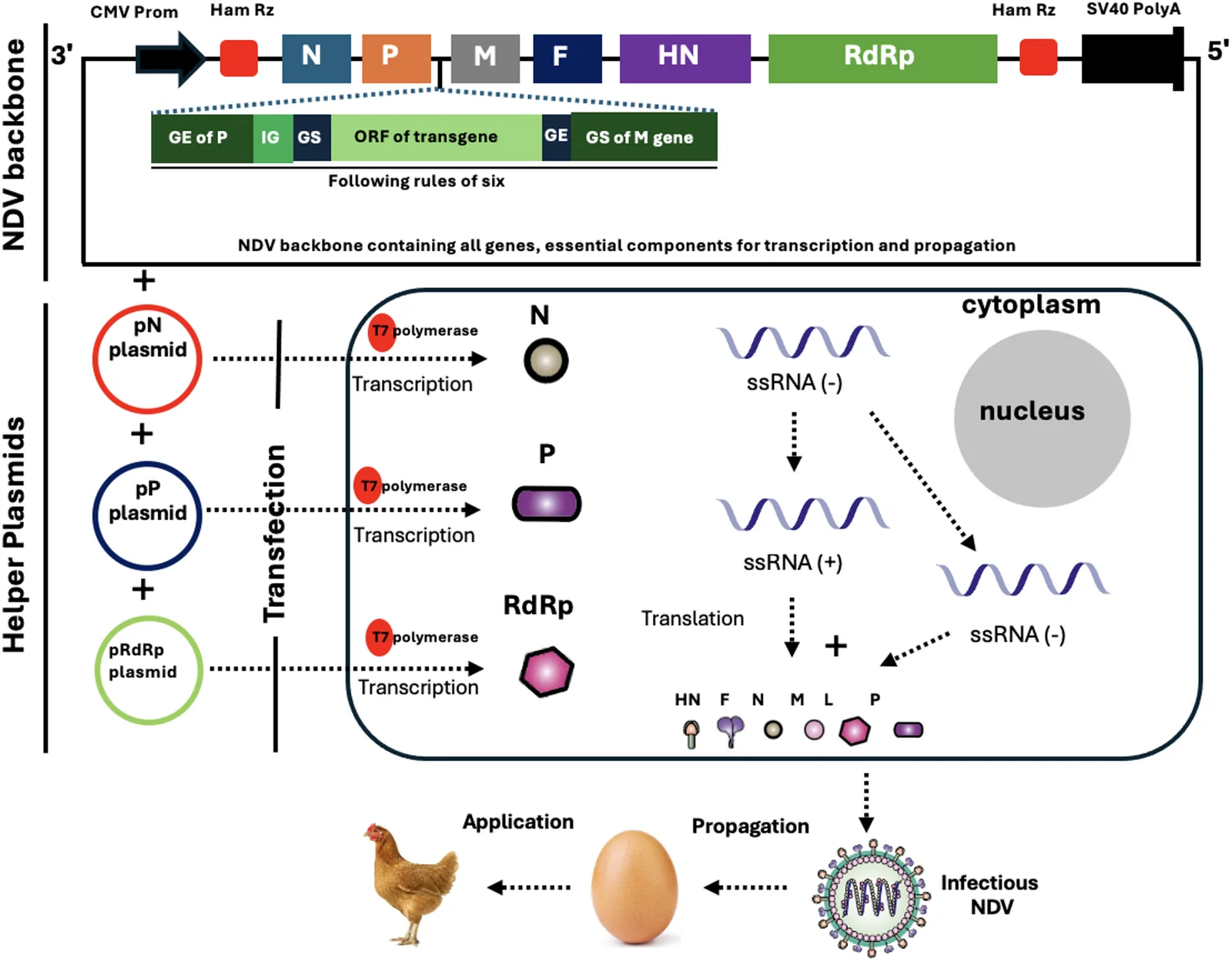
Genomic organization and the protocol to rescue rNDV using reverse genetics. The genome of NDV consists of six genes (N, P, M, F, HN and RdRP/L) and are flanked by hammer head ribozymes. The 3’ end is CMV promoter to initiate transcription of all viral genes through start-stop mechanisms and termination of transcription by the SV40 PolyA tail. Transfection of NDV backbone (consisting of all genes and transcription components) and three helper plasmids (N, P and RdRP/L) for transcription complex. The genomic replication product along with expressed structural proteins are assembled into virions and lead to rescue of virus. The rescued rNDV is then propagated in chicken embryonated eggs before formulation and application for immunization purposes

Reverse genetics has fundamentally transformed the field of NDV vaccinology, shifting the paradigm from empirical attenuation to precise, rational design. This powerful technique enables the *de novo* rescue of infectious virus from cloned complementary DNA (cDNA), thereby allowing researchers to engineer the viral genome with unprecedented precision for safety, efficacy, and novel functionality. The core system involves the construction of a full-length antigenomic cDNA clone and its co-transfection with supporting plasmids expressing the essential NP, P, and L proteins to reconstitute infectious particles [58] (Fig. 3). This foundational capability serves as the engine for several advanced vaccine strategies. One of the most impactful applications is the targeted attenuation of virulent strains to create inherently safer vaccine candidates. This is achieved primarily by modifying the key molecular determinant of virulence: the F protein cleavage site. Through reverse genetics, the polybasic motif characteristic of velogenic strains can be precisely replaced with the monobasic motif of lentogenic strains. This single change effectively converts a lethal pathogen into a replication-competent, attenuated vaccine while preserving the antigenic backbone of the circulating virulent genotype, a strategy that promises improved immune matching compared to historical vaccine strains [149]. Beyond attenuation, the technology is indispensable for creating vaccines compatible with a differentiating infected from vaccinated animals (DIVA) strategy. By deleting a small immunogenic region from a non-essential protein or inserting a foreign epitope tag, researchers can generate marker vaccines. Vaccinated animals produce a distinct antibody profile, allowing for serological distinction from naturally infected birds using companion diagnostic tests, a critical feature for disease surveillance and eradication programs [21]. Furthermore, reverse genetics is the essential tool for developing sophisticated recombinant vectored vaccines. It allows for the precise insertion of foreign gene expression cassettes into the NDV genome, enabling the creation of multivalent vaccines that confer concurrent protection against NDV and other major poultry pathogens (as detailed in Section “Recombinant vector vaccines”) [40, 92]. The technology also facilitates advanced research and development tools, such as the insertion of reporter genes [21] to create viruses that allow for real-time tracking of replication dynamics, high-throughput antiviral screening, and quantitative assessment of vaccine take in vivo [118]. Perhaps most significantly for contemporary control challenges, reverse genetics enables the design of genotype-matched vaccines. This involves generating chimeric viruses where the antigenic F and HN genes of a classical, safe vaccine backbone are replaced with those from a currently circulating virulent genotype. The resulting vaccine combines proven safety and growth characteristics with the antigenic profile of the field virus, a direct approach to closing the antigenic gap, improving cross-protection, and reducing viral shedding in endemic regions [117]. In summary, reverse genetics provides a versatile and precise platform to custom-build next-generation NDV vaccines that address the specific limitations of conventional platforms, paving the way for more effective and sustainable disease control.

### HVT-based vaccines for NDV

The herpesvirus of turkeys (HVT; Meleagrid herpesvirus 1), a naturally avirulent member of the family Herpesviridae, subfamily Alphaherpesvirinae, genus Mardivirus, represents one of the most commercially successful and widely adopted viral vector platforms in modern poultry vaccinology. Its success is attributed to a unique biological profile that addresses several critical limitations of conventional NDV vaccines. Recombinant HVT (rHVT) engineered to express the immunogenic NDV F protein, commercialized as products like Innovax-ND, has revolutionized vaccination strategies, particularly in commercial broilers and breeders [28]. The efficacy of rHVT-NDV stems from HVT’s ability to establish a persistent, lifelong latent infection in the host without causing disease. Following administration, the virus replicates lytically, disseminates, and, consistent with the behaviour of other alphaherpesviruses, establishes latency predominantly in neural tissue, alongside a lifelong reservoir in circulating lymphocytes. During this persistent infection, the inserted NDV F gene is continuously expressed, providing a sustained antigenic stimulus. This results in the gradual development of robust, long-lasting immunity that matures over several weeks, offering durable protection throughout the bird’s life without the need for revaccination [114]. A paramount advantage is its complete resistance to neutralization by maternal antibodies (MDAs). Because HVT is antigenically distinct from NDV, circulating anti-NDV MDAs do not interfere with the vector’s replication or the immune response to the vectored F antigen. This eliminates the “window of susceptibility” seen with live NDV vaccines, allowing for effective immunization at the day of hatch [39]. The administration route further enhances its practicality. rHVT-NDV is typically delivered *in ovo* to 18-day-old embryonated eggs using automated multi-egg injectors or subcutaneously to day-old chicks in the hatchery. This enables 100% vaccine coverage with minimal bird handling and stress, seamlessly integrating into high-throughput commercial hatchery operations. The single-shot solution provides concurrent protection against both Marek’s disease (via the HVT backbone) and ND, simplifying complex vaccination schedules [36]. While highly effective, the platform has nuances. Immunity is not immediate but develops over 3–4 weeks post-vaccination, requiring that birds are protected from early field virus exposure by other means, such as biosecurity or the use of a live NDV vaccine primer. Additionally, the cell-associated nature of the vaccine requires careful handling of the frozen cell-associated vaccine product. Despite these considerations, the rHVT platform exemplifies a successful transition from conventional to rationally designed recombinant vaccines, offering a safe, logistically efficient, and highly effective tool that has become a cornerstone of integrated health management in the global poultry industry.

## Delivery systems and formulation innovations

### Mucosal delivery approaches (Ocular, Nasal, Oral)

Mucosal vaccination aims to induce immune protection directly at the primary sites of NDV entry and replication, the respiratory and digestive tracts. This approach is particularly effective for live attenuated and some recombinant vector vaccines, as it mimics the natural route of infection, stimulating both local and systemic immunity. Ocular and intranasal delivery are considered the gold standard for priming immunity with live vaccines. These methods deposit the vaccine directly onto the ocular conjunctiva and nasal mucosa, areas rich in mucosa-associated lymphoid tissue (MALT). This facilitates efficient antigen uptake, leading to the robust production of secretory IgA (sIgA) and the establishment of tissue-resident memory T cells at the portal of entry, providing an immediate first line of defense [68]. Fine spray vaccination, often used in hatcheries for day-old chicks, ensures uniform droplet size for deep respiratory tract deposition. Oral vaccination, typically administered via drinking water, offers unparalleled convenience for mass application in rearing and laying flocks, minimizing bird stress and labor costs. However, its major drawback is inconsistent vaccine take. Efficacy can be severely compromised by factors such as chlorine or metal ions in the water that inactivate the virus, variable water consumption among birds, and the degradation of the vaccine virus by the low pH and digestive enzymes in the proventriculus and gizzard before it can infect the intestinal lymphoid tissues [96]. To improve oral efficacy, research focuses on enteric protection strategies, including the use of virus encapsulation in acid-resistant polymers or the selection of vaccine strains with greater enteric stability. Despite its challenges, oral delivery remains a vital tool for booster vaccinations in large flocks where individual handling is impractical.

### Nanoparticle and liposomal delivery platforms

Nanotechnology has emerged as a pivotal tool to overcome the inherent limitations of next-generation vaccine platforms, particularly subunit, VLP, and nucleic acid vaccines. Nanoparticle and liposomal delivery systems are engineered to enhance antigen stability, immunogenicity, and targeted delivery, bridging the gap between novel antigen design and practical efficacy [17]. These platforms function through several key mechanisms. First, they provide robust protection for encapsulated or adsorbed antigens against enzymatic degradation in physiological environments, a critical factor for oral or mucosal delivery. Second, they enable controlled or sustained antigen release, prolonging exposure to the immune system and potentially reducing the number of required doses. Most importantly, their particulate nature (typically 20–200 nm) and surface properties can be engineered to mimic pathogens, promoting efficient uptake by antigen-presenting cells (APCs) such as dendritic cells and macrophages. This direct targeting to APCs enhances antigen processing and presentation, leading to stronger and more balanced humoral and cell-mediated immune responses [156]. Liposomes, spherical vesicles with phospholipid bilayers, are a well-established delivery system. They can encapsulate hydrophilic antigens in their aqueous core and hydrophobic antigens within their lipid membranes. Cationic liposomes are particularly effective for complexing with negatively charged nucleic acids, forming lipoplexes that facilitate cellular entry and have been successfully used in preclinical NDV mRNA vaccine studies [20]. Polymeric nanoparticles, such as those made from poly(lactic-co-glycolic acid) (PLGA) or chitosan, offer tunable degradation rates and surface functionalization. Chitosan nanoparticles, due to their mucoadhesive properties, are especially promising for mucosal vaccination, adhering to the mucus layer and enhancing antigen uptake across respiratory or intestinal epithelium [126]. Advanced “smart” nanoparticles can be functionalized with ligands to actively target receptors on APCs. Furthermore, they can be co-loaded with antigens and immunostimulatory adjuvants within the same particle, ensuring both components are delivered to the same cell, thereby synergistically potentiating the immune response [147]. While challenges in scalable, cost-effective good manufacturing practice (GMP) production and long-term stability remain, nanoparticle and liposomal platforms hold immense potential for formulating stable, single-dose, and mucosally administered next-generation NDV vaccines.

### Adjuvants used in NDV vaccines

Adjuvants are critical components that enhance and direct the immune response to vaccine antigens, particularly for platforms like inactivated and subunit vaccines that possess lower intrinsic immunogenicity. The classic and most effective adjuvant for inactivated NDV vaccines is the oil-based water-in-oil (W/O) emulsion. These formulations, typically using mineral oil, function by creating a persistent antigen depot at the injection site. This depot facilitates the slow release of antigen over an extended period and stimulates a localized inflammatory response rich in immune cells. The primary immunological outcome is a strong, Th2-biased humoral response, characterized by the production of high and sustained titers of virus-neutralizing IgG antibodies, which is the fundamental goal of an inactivated vaccine booster 3) [43]. Beyond these traditional emulsions, the development of next-generation NDV vaccines has driven research into novel adjuvants designed to elicit a more comprehensive immune profile. The goal is to stimulate not only robust antibodies but also potent cell-mediated immunity (CMI), which is crucial for cross-protection and clearing infected cells. This new generation includes immunomodulators such as recombinant chicken cytokines. Molecules like IL-12 and IFN-γ are administered with vaccines to promote a Th1-type immune environment, directly enhancing the development of antigen-specific CD8 + cytotoxic T lymphocytes (CTLs) that are vital for effective viral clearance [158]. Another promising class comprises synthetic CpG oligodeoxynucleotides (ODNs), which mimic bacterial DNA and act as agonists for Toll-like receptor 9 (TLR9) in birds. Their engagement potently activates innate immune pathways, leading to a pro-inflammatory cytokine milieu and improved antigen presentation, thereby amplifying both antibody and CMI responses [89]. Furthermore, naturally derived saponins like Quil-A are used to form sophisticated structures called ISCOMs. ISCOMs serve a dual function - they enhance antigen delivery to antigen-presenting cells while also providing intrinsic immunostimulation, effectively promoting a balanced humoral and Th1/CTL response, making them highly suitable for subunit and VLP vaccine platforms [64]. The strategic selection and formulation of these advanced adjuvants are pivotal for tailoring immune outcomes and maximizing the efficacy of modern NDV vaccine candidates.

### Mass-immunization technologies (Spray, Drinking water, In-Ovo)

The economic viability of poultry production is fundamentally dependent on efficient, large-scale vaccination methods that minimize bird handling, labor costs, and stress while ensuring uniform flock immunity. Several mass-immunization technologies have been developed to meet this critical need; each suited to specific stages of production and vaccine types. For live attenuated vaccines in growing pullets, layers, and breeder flocks, spray vaccination is a predominant method. This technique involves generating an aerosol of the vaccine using specialized equipment, allowing for the simultaneous vaccination of thousands of birds in a house. Coarse spray is primarily used for older birds, targeting the upper respiratory tract and ocular mucosa. For day-old chicks, fine spray can achieve deeper respiratory deposition. Spray vaccination effectively stimulates local mucosal immunity and is highly efficient, though its success depends on proper equipment calibration, environmental conditions, and uniform bird exposure [96]. Drinking water vaccination is another cornerstone of mass application, particularly for booster doses. Its principal advantage is extreme convenience, as it leverages the birds’ natural drinking behavior. However, its major drawback is inconsistent vaccine take, influenced by variable water consumption, water quality, and the timing of water withdrawal prior to vaccination. Careful management is required to neutralize chlorine and ensure rapid, uniform consumption to maximize efficacy. The most technologically advanced and precise mass application method is *in ovo* vaccination. This involves the automated injection of vaccine into the amniotic fluid of 18-day-old embryonated eggs using sophisticated multi-egg injectors. This method, now standard in the global broiler industry, offers unparalleled advantages: it ensures 100% vaccine coverage with absolute consistency, eliminates post-hatch handling stress, and allows for the very early induction of immunity. Vaccines administered *in ovo*, such as recombinant HVT-NDV, begin stimulating the developing immune system before hatch, leading to more uniform and robust protection as chicks emerge. This technology seamlessly integrates vaccination into the hatchery workflow, representing the pinnacle of automation and precision in preventive poultry health management [1].

## Immunological considerations influencing vaccine efficacy

### Humoral and cellular immune responses

Effective protection against NDV is a product of coordinated humoral and cellular immune arms. The humoral response, characterized by the production of neutralizing antibodies, is the primary and most easily measured correlate of protection. Hemagglutination inhibition (HI) antibody titers strongly correlate with resistance to clinical disease and mortality; titers at or above a threshold (typically 4 log₂ or 1:16) are considered protective. These antibodies, primarily targeting the surface F and HN glycoproteins, function by blocking viral attachment and entry into host cells, thereby preventing viremia and systemic spread [101]. However, sterilizing immunity, which prevents infection and shedding, requires a robust cell-mediated immune (CMI) response. This arm, mediated by CD4 + T helper cells and, crucially, CD8 + cytotoxic T lymphocytes (CTLs), is responsible for identifying and eliminating virus-infected host cells. CTLs recognize viral peptides presented on major histocompatibility complex (MHC) class I molecules, a process that is particularly effective when vaccines facilitate endogenous antigen expression, as seen with live attenuated, viral vector, and nucleic acid platforms. A potent CMI response is essential for clearing established infection, limiting viral replication in tissues, and providing cross-protection against antigenically variant strains, as T cell epitopes are often more conserved than the B cell epitopes targeted by antibodies [77]. Therefore, an ideal NDV vaccine should be designed to stimulate a balanced and synergistic response: high-titer neutralizing antibodies to prevent initial infection and severe disease, coupled with a strong CTL response to eradicate any breakthrough infection and minimize viral shedding.

### Duration and breadth of protection

The practical value of an NDV vaccine is defined not only by the strength of the immune response it elicits but also by its longevity and scope. The duration of protection varies significantly between vaccine platforms, primarily dictated by the nature of the antigenic stimulus and the type of immunological memory established. Live attenuated vaccines (LAVs) typically induce rapid immunity that manifests within days, a critical feature for outbreak response. However, this protection may wane more quickly, especially in the face of high maternal antibody interference or intense field virus challenge, necessitating booster vaccinations. In contrast, inactivated vaccines, when adjuvanted with oil emulsions, and live viral vector vaccines like rHVT-ND, are renowned for providing long-lasting immunity. The depot effect of oil adjuvants ensures prolonged antigen exposure, while persistent vectors like HVT offer continuous, low-level antigen expression, both strategies promoting durable memory B and T cell responses that can protect birds throughout a full laying or breeding cycle [137]. A more formidable and persistent challenge is the breadth of protection, the ability of a vaccine based on one viral genotype to confer robust immunity against antigenically distinct, heterologous genotypes. This is a central limitation of conventional vaccines. While they often protect against severe disease and mortality from heterologous challenges, they frequently fail to prevent infection, subsequent viral replication, and shedding. This “leaky” immunity allows virulent field viruses to circulate silently in vaccinated populations, driving viral evolution and complicating eradication efforts [60]. The breadth of protection is intimately linked to the degree of genetic and antigenic divergence between the vaccine strain and the circulating field virus. The global dominance of genotypes like V, VII, and XIII, which are phylogenetically distant from the classical genotype II vaccine strains, has exacerbated this challenge, underscoring the need for genotype-matched vaccines or platforms capable of inducing broad, universal responses targeting conserved viral epitopes.

### Mucosal immunity and local protection

The respiratory and intestinal tracts serve as the primary portals for NDV entry and initial replication. Therefore, establishing effective immune surveillance at these mucosal surfaces is paramount for achieving sterilizing immunity and interrupting transmission cycles. Mucosal immunity, characterized by the presence of secretory IgA (sIgA) and tissue-resident memory T cells, provides a crucial first line of defense by neutralizing the virus at the site of infection before it can establish a systemic foothold [30].The induction of potent mucosal immunity is highly dependent on both the vaccine platform and the route of administration. Live attenuated vaccines and certain replicating viral vectors are uniquely effective at stimulating this local response because they replicate within mucosal tissues, mimicking natural infection. When these vaccines are delivered via mucosal routes, such as ocular drop, nasal spray, or, to a lesser extent, oral administration, they directly interact with the nasopharynx-associated lymphoid tissue (NALT) and gut-associated lymphoid tissue (GALT). This interaction triggers the activation of mucosal B cells, leading to the production of pathogen-specific sIgA, which can bind and neutralize virus particles in the respiratory secretions and intestinal lumen [52]. Furthermore, this localized infection promotes the generation of resident memory T cells that can mount rapid recall responses upon subsequent viral exposure.

Vaccines that are administered parenterally primarily stimulate strong systemic immunity but generate limited mucosal sIgA. Consequently, while they protect against systemic disease, they may be less effective at preventing initial infection and subsequent viral shedding from the respiratory or digestive tracts. For comprehensive control, vaccination programs often employ a prime-boost strategy that combines a mucosal prime with a live vaccine to establish local immunity, followed by a systemic booster to elevate and prolong circulating antibody titers, thereby addressing both immediate viral blockade and long-term systemic protection [87].

It is important to acknowledge that the evidence base underpinning this model remains incomplete. A direct, quantitative correlation between specific sIgA titres and protection against NDV challenge has not been firmly established in the published literature, and sIgA measurement itself lacks standardization across studies, with variable sampling sites, assay platforms, and normalization methods limiting cross-study comparison. Characterization of avian tissue-resident memory T cells is likewise still under development, and direct evidence that Trm frequency or phenotype predicts heterologous protection remains sparse. The relative contributions of sIgA, mucosal IgG, lysozyme, complement, and innate pattern-recognition receptors to overall mucosal protection are therefore best regarded as an active area of investigation rather than a settled mechanism.

### Immune interference and maternal antibodies

A significant biological hurdle in early-life vaccination against NDV is the presence of Maternally Derived Antibodies (MDA). These antibodies are transferred from vaccinated hens to their progeny via the yolk, providing passive protection during the first weeks of life. However, they also pose a major obstacle to active immunization. Circulating MDA can bind to and neutralize live attenuated vaccine virus before it can infect cells and replicate sufficiently to stimulate a robust adaptive immune response. This interference often results in vaccination failure, creating a vulnerable period known as the “window of susceptibility”, when MDA levels are too low to protect against field virus but still high enough to block vaccine take [15]. Overcoming MDA interference is critical for effective vaccination programs. Two primary strategies are employed. The first is careful timing of vaccination, which involves delaying the administration of live vaccines until MDA titers have naturally decayed to a sub-neutralizing level. This requires knowledge of MDA half-life and often involves serological monitoring to determine the optimal vaccination window, a practice that can be challenging to implement consistently in field conditions. The second, more robust strategy is the use of MDA-resistant vaccine platforms. Recombinant viral vector vaccines, such as those based on the herpesvirus of turkeys (rHVT-ND), are not antigenically related to NDV. Therefore, anti-NDV MDA do not recognize or neutralize the vector, allowing it to replicate unimpeded and express the NDV F protein, successfully priming the chick’s own immune system from day one [105]. Other approaches include using higher-titer live vaccines to “overwhelm” low levels of MDA or employing inactivated vaccines for the initial dose, as their immunogenicity is not neutralized by MDA, though they require a prime from a live vaccine to be fully effective. Successfully navigating MDA interference is fundamental to ensuring uniform early protection and closing the immunity gap in young poultry.

## Current challenges in NDV vaccine development

### Antigenic diversity and emergence of new genotypes

The dynamic evolution of NDV represents one of the most formidable obstacles to sustained vaccine efficacy. The virus exhibits substantial genetic plasticity, leading to the continuous emergence and global spread of novel genotypes and sub-genotypes. While classical lentogenic vaccine strains like LaSota (genotype II) have been used successfully for decades, their phylogenetic distance from currently dominant virulent genotypes, such as VII.1.1 in Asia and Africa and XIII.2.1 in parts of Asia and the Indian subcontinent, creates a concerning antigenic mismatch [82]. This genetic divergence can translate into antigenic drift, where key neutralizing epitopes on the F and HN glycoproteins evolve sufficiently to reduce the binding affinity of antibodies induced by the vaccine strain. The consequence is often leaky immunity: vaccinated birds are protected from severe clinical disease and mortality but are not fully prevented from infection, viral replication, and subsequent shedding of the challenge virus [100]. This incomplete protection allows virulent field strains to circulate silently in vaccinated populations, facilitating further viral evolution, perpetuating outbreaks, and complicating eradication efforts. The recurring reports of ND outbreaks in flocks vaccinated with genotype II-based vaccines in regions where genotype VII is endemic provide compelling field evidence of this limitation [153]. This challenge underscores the urgent need for surveillance-informed, genotype-matched vaccine strategies and the exploration of vaccines targeting more conserved viral epitopes to achieve broader cross-protection.

### Incomplete cross-protection

A critical and well-documented limitation of conventional NDV vaccines, particularly those based on classical genotypes, is their incomplete or “leaky” cross-protection against heterologous viral strains. While these vaccines typically provide robust protection against severe clinical disease and mortality, often the primary metric for vaccine licensure, they frequently fail to induce sterilizing immunity [67]. This means that vaccinated birds, when challenged with a genetically divergent field virus, can become productively infected. They may exhibit minimal or no clinical signs but can still replicate the challenge virus and shed it into the environment via respiratory secretions and feces [34]. This phenomenon has profound implications for disease control and epidemiology. The inability to prevent shedding allows silent circulation of virulent viruses within vaccinated populations. These viruses can then spread to unvaccinated or susceptible birds, including wild avian species, and continue to evolve under immune pressure. This persistent environmental viral load undermines eradication efforts, complicates biosecurity, and can lead to sporadic outbreaks even in well-vaccinated flocks [4]. The gap between clinical protection and virological protection highlights those traditional correlates of immunity, such as HI titers, may not fully predict a vaccine’s ability to block transmission. Overcoming this requires vaccines that elicit not only high-titer neutralizing antibodies but also potent mucosal immunity and cross-reactive T-cell responses capable of rapidly eliminating infected cells before significant viral progeny are produced.

### Cold-chain and logistics in low-resource settings

The practical implementation of vaccination programs is often critically constrained by logistical and infrastructural limitations, particularly in low-resource and remote settings where village poultry production is predominant. Most commercial live attenuated NDV vaccines are lyophilized to extend shelf life but remain thermolabile, requiring an unbroken cold chain (2–8 °C) from manufacturer to point of use. Maintaining this cold chain in regions with limited refrigeration, unreliable electricity, and challenging transportation networks is often impossible, leading to progressive loss of vaccine potency and widespread vaccination failure [12]. These logistical hurdles contribute significantly to the endemic persistence of ND in many developing countries. To address this, concerted efforts have been made to develop thermostable vaccine strains. The most notable example is the I-2 strain, selected specifically for its ability to retain potency for extended periods at ambient temperatures [14]. While thermostable vaccines like I-2 represent a major advancement for village poultry health, challenges remain in scaling up their production, ensuring consistent quality, and integrating them into sustainable delivery systems. Beyond thermostable formulations, other promising solutions include nanoparticle-based delivery systems that protect antigens from degradation, and solid-dose or film-based vaccines that eliminate liquid handling. Ultimately, overcoming the cold-chain barrier is not merely a technical issue but a necessary step for equitable disease control, enabling effective vaccination to reach the millions of smallholder farmers who are most vulnerable to Newcastle disease.

### Safety concerns and reversion risks

While LAVs are invaluable tools, they are not without inherent safety concerns that influence their use and regulatory approval. A primary theoretical risk, though considered very low for well-characterized lentogenic strains, is reversion to virulence. This could occur through cumulative point mutations or, more likely, through recombination with circulating field viruses, potentially restoring a polybasic F protein cleavage site. Although rare, the potential consequences are severe, justifying stringent biocontainment during production and ongoing surveillance [69]. More immediate and practical safety issues involve vaccine-induced pathogenicity. Even lentogenic strains can cause mild to moderate respiratory reactions (coughing, rales), which can predispose birds to secondary bacterial infections like E. coli and lead to economic losses in performance. In very young chicks, embryos, or immunocompromised birds, these reactions can be more severe. Mesogenic strains, which are more immunogenic but also more pathogenic, pose a higher risk. Their capacity to cause clinical disease, reduce egg production, and spread to susceptible contacts has led to restrictions or outright bans on their use in many countries, limiting their role in control programs despite their historical use as boosters. These safety profiles underscore the advantage of next-generation platforms, such as inactivated, subunit, VLP, and nucleic acid vaccines where the replication risks are eliminated altogether. Additionally, rationally designed recombinant vectors which are well-characterized, and are non-pathogenic backbones offer safer utilization of vaccines against NDV.

### Differentiating infected from vaccinated animals (DIVA) limitations

The inability to distinguish between vaccine-induced and natural infection-derived immunity represents a major epidemiological constraint in NDV control programs, particularly in regions pursuing eradication or proof of disease-free status. Conventional live attenuated and inactivated vaccines stimulate antibodies against all major viral proteins, creating an identical serological profile to that produced by wild-type infection. This lack of a DIVA capability severely hampers effective surveillance, outbreak investigation, and monitoring of viral circulation in vaccinated populations [146]. Without a DIVA strategy, serological testing becomes virtually useless for surveillance in vaccinated flocks, as positive results cannot discern whether they indicate successful vaccination, a past field infection, or an active outbreak. This blind spot allows the virus to circulate undetected, undermining control efforts and complicating international trade, which often requires evidence of freedom from infection rather than just vaccination. The development and implementation of companion DIVA tests alongside novel vaccines is therefore critical. Successful DIVA strategies typically involve vaccines that lack one or more immunogenic proteins present in the wild-type virus. For example, a vaccine expressing only the F protein (and not the NP or M proteins) enables the use of an ELISA-based detection antibodies to the missing protein(s) as a marker of natural infection [40]. Alternatively, recombinant vaccines can incorporate an antigenic marker, such as a unique epitope tag. The widespread adoption of such integrated vaccine-DIVA systems is essential for transitioning from mere clinical disease control to true epidemiological monitoring and progressive eradication of ND.

It is important to note that no live attenuated or inactivated NDV vaccine currently in commercial use in endemic regions carries a validated DIVA marker; companion DIVA diagnostics remain largely confined to recombinant vector or subunit platforms that are themselves not yet widely deployed in these settings. DIVA testing also carries a substantial cost and infrastructure penalty relative to routine serology: blocking ELISA or other marker-specific assays typically cost in the order of US$5–20 per test and require calibrated plate readers and trained laboratory personnel, compared with approximately US$1–2 per test and comparatively simple equipment for conventional haemagglutination-inhibition (HI) assays. Consequently, DIVA capability delivers the greatest practical value in settings with well-resourced surveillance systems, regulatory frameworks built around disease-free status, and low background field-virus circulation; its near-term utility in endemic low-resource regions, where NDV continues to circulate widely and diagnostic capacity is limited, is correspondingly constrained [60].

## Field application and real-world performance

### Vaccination schedules across production systems

Optimal NDV vaccination is not a one-size-fits-all approach but is meticulously tailored to the specific production cycle, purpose, and risk profile of the flock. In broiler production, where the lifecycle is short (5–7 weeks), the goal is to establish rapid and uniform protection with minimal bird handling. Schedules are highly streamlined, often relying on a single administration of a live vaccine via coarse spray at the day of hatch or, increasingly, the use of *in ovo* vaccination with a recombinant vector like rHVT-ND. In some higher-risk regions, a live vaccine spray prime may be followed by a booster via drinking water around 14–21 days of age [50, 111]. For layer and breeder flocks, which have a productive lifespan exceeding 60 weeks, the strategy is fundamentally different and more complex. The objective is to induce and maintain high, persistent levels of protective antibodies (both systemic and maternal) throughout the laying period. This requires a multi-phase vaccination program. It typically begins with a series of live vaccine primes (via spray or eye drop) during the rearing period (at day-old, 2–3 weeks, and 6–8 weeks) to build a strong cellular and mucosal immune foundation. This is followed by one or more injections of inactivated, oil-adjuvanted vaccines at the point of lay (16–18 weeks) to elicit a massive and durable antibody response. Breeders may receive additional inactivated boosters during the laying period to ensure the transfer of high, uniform maternal antibody titers to their progeny, protecting chicks during their first critical weeks [49]. These intensive schedules are essential to close the immunity gap and prevent catastrophic drops in egg production or quality due to mid-cycle NDV challenges.

### Outbreak case studies

Despite widespread vaccination, ND outbreaks in vaccinated flocks remain a recurrent global issue, serving as critical real-world case studies that expose the limitations of current control strategies. These outbreaks are often multifactorial, with investigations typically revealing a confluence of virological, managerial, and immunological failures. A persistent theme is genotype mismatch and antigenic divergence. Numerous well-documented outbreaks in Asia, Africa, and the Middle East have occurred in flocks regularly vaccinated with the LaSota (genotype II) strain but challenged with virulent genotype VII field viruses. For instance, severe outbreaks in Indonesia and China in vaccinated commercial flocks were definitively linked to genotype VII.1.1 viruses. While mortality was often reduced compared to unvaccinated controls, significant clinical disease, egg production drops, and high viral shedding were observed, demonstrating the incomplete cross-protection afforded by heterologous vaccines [140]. Similar scenarios have been reported with other genotypes, such as XIII in India [53]. Beyond viral evolution, programmatic failures are frequently implicated. These include breakdowns in the cold chain leading to administration of inactivated vaccine, improper vaccine handling or administration (incorrect dilution, inadequate water withdrawal for drinking water vaccines), and failure to account for maternal antibody interference, resulting in uneven vaccine “take” within a flock. Furthermore, concurrent immunosuppression due to infections like Infectious Bursal Disease (IBD), Marek’s disease, or mycotoxins can severely blunt the vaccine-induced immune response, rendering even well-vaccinated birds susceptible [136]. These case studies underscore that vaccine failure is often a systems failure, highlighting the need for integrated health management that combines genotype-relevant vaccines, flawless logistics, robust biosecurity, and control of immunosuppressive agents.

### Monitoring vaccine take and seroconversion

The implementation of a vaccination program does not guarantee its success; therefore, systematic monitoring is a critical component of any effective ND control strategy. This post-vaccination evaluation serves to verify vaccine efficacy, identify program flaws, and inform timely corrective actions. Serological monitoring is the most common and standardized approach. The HI test is the gold standard for assessing seroconversion and measuring the level of protective humoral immunity. By tracking HI antibody titers at strategic intervals (2–4 weeks post-vaccination), producers can determine if the flock has achieved and maintained protective titers (typically ≥ 4 log₂). This is especially crucial for breeder flocks to ensure adequate maternal antibody transfer. The pattern of titers, such as a failure to seroconvert, low mean titers, or high individual variability, can diagnose problems like vaccine inactivation, improper administration, or overwhelming maternal antibody interference [31]. Beyond serology, virological and molecular methods provide direct evidence of vaccine “take.” For live vaccines, reverse transcription-polymerase chain reaction (RT-PCR) or virus isolation can be used to detect vaccine virus shedding in oropharyngeal or cloacal swabs collected 3–7 days post-vaccination. The absence of detectable vaccine virus in a sample of birds indicates a failure in vaccine delivery or viability. Furthermore, clinical observation for expected, mild post-vaccination reactions can serve as a crude but immediate field indicator of vaccine replication. A complete absence of reaction might signal a problem with the vaccine or its administration. Together, these monitoring tools form an essential feedback loop, transforming vaccination from a routine task into a measurable and manageable component of flock health.

### Biosecurity integration with vaccination programs

A foundational principle of effective ND control is that vaccination and biosecurity are synergistic, non-interchangeable components of a holistic health strategy. Vaccination prepares the host to resist infection, while biosecurity aims to prevent the pathogen from reaching the host in the first place. Relying on vaccination alone is a high-risk strategy, as it places continuous immune pressure on the virus and can lead to the silent circulation of field strains, as previously discussed. A comprehensive program therefore integrates strict biosecurity measures to minimize the initial viral challenge pressure. This includes controlling access to farms (via fencing, disinfectant footbaths, and visitor logs), ensuring vehicles and equipment are cleaned and disseminated, implementing robust rodent and insect control, and maintaining an “all-in/all-out” production system with thorough cleaning and disinfection between flocks. For backyard and village systems, this may involve basic measures like housing birds at night to protect from wild birds and avoiding the introduction of birds from markets without quarantine [24, 102]. This reduced environmental viral load allows the vaccination program to perform optimally. Vaccines are most effective when birds are exposed to a low, manageable dose of virus; overwhelming exposure can breach even good immunity. Furthermore, high biosecurity helps protect young chicks during the window before vaccine-induced immunity is fully functional. Conversely, vaccination provides a critical “safety net” by establishing herd immunity, reducing the probability of virus spread if a biosecurity breach occurs. This integrated approach using biosecurity as the first line of defense and vaccination as the essential second layer creates a resilient system that is far more capable of sustaining poultry health and productivity than either component alone.

## Regulatory and manufacturing considerations

### Global regulatory landscape

The pathway to licensure for an NDV vaccine is defined by a multi-faceted regulatory environment that balances international harmonization with national sovereignty. Globally, the WOAH sets foundational standards through its Manual of Diagnostic Tests and Vaccines for Terrestrial Animals. These guidelines outline the essential criteria for vaccine quality, including principles for demonstrating safety, potency, efficacy, and purity [5]. However, ultimate authority rests with national regulatory agencies, each with its own specific legal and technical requirements. In the United States, the Center for Veterinary Biologics (CVB) within the USDA oversees a rigorous process involving detailed product characterization, target animal safety studies, and efficacy trials. In the European Union, the European Medicines Agency (EMA) provides scientific evaluation, with marketing authorization granted by the European Commission. Other major poultry-producing countries, such as Brazil, China, and India, have their own competent authorities with distinct dossier requirements and review processes [132]. This regulatory fragmentation poses a significant challenge for manufacturers seeking global market access, often necessitating costly and time-consuming parallel submissions and compliance with divergent testing protocols. The emergence of novel platforms, such as mRNA and plant-derived VLPs, further tests these regulatory frameworks, requiring adaptations to evaluate products that do not fit traditional paradigms.

### Challenges in large scale production

Translating a successful vaccine candidate into a commercially viable product that can meet global demand presents significant manufacturing hurdles. Scalability, the ability to increase production volume efficiently, consistently, and cost-effectively is a critical determinant of a vaccine’s real-world impact. A primary bottleneck for conventional live attenuated and inactivated vaccines is their reliance on SPF embryonated chicken eggs. This production system faces inherent limitations: the finite and costly supply of SPF eggs, the biological variability between eggs, and the lengthy incubation period. Scaling up to meet sudden surges in demand, such as during a widespread outbreak, is slow and logistically challenging, creating potential vaccine shortages [22]. Next-generation platforms offer promising pathways to more scalable and controllable manufacturing. Cell culture-based production using continuous avian cell lines (DF-1) provides a more homogeneous and scalable substrate than eggs, enabling production in large-volume bioreactors with greater process standardization. For nucleic acid vaccines, manufacturing is fundamentally chemical and cell-free. mRNA vaccines, in particular, are produced via in vitro transcription reactions that are highly scalable, rapid, and amenable to platform-based production where only the DNA template sequence needs to be changed for different targets [138]. Similarly, subunit and VLP vaccines produced in microbial or plant-based systems can be scaled in industrial fermenters or through agricultural cultivation, offering potentially lower-cost alternatives. However, each advanced platform brings its own scaling challenges, such as optimizing complex purification processes for VLPs, ensuring the stability of lipid nanoparticles for mRNA, or achieving high-yield expression of properly folded proteins in novel systems. Overcoming these bioprocessing challenges is essential to transform innovative vaccine science into a sustainable and responsive supply for the global poultry industry.

### Quality control and standardization

Maintaining unwavering consistency across vaccine batches is the cornerstone of manufacturing integrity and field efficacy. Rigorous Quality Control (QC) protocols are enforced to ensure every released vial meets predefined specifications for safety, potency, purity, and identity, thereby building trust in vaccination programs. Potency testing, a central QC requirement, varies by platform. For live attenuated vaccines, potency is quantified as the infectious virus titer, typically measured in 50% egg infective doses (EID₅₀) per dose. This must fall within a strict, validated range to guarantee adequate immunogenic stimulus. For inactivated vaccines, potency is correlated with antigen content, often assessed through immunological assays such as ELISA to quantify key proteins (F/HN), alongside physical measurements of antigen integrity. Ultimately, the immunogenic potency of both types is confirmed through in vivo challenge studies in SPF chickens, where vaccinated groups must demonstrate a statistically significant level of protection against a standardized virulent NDV challenge compared to unvaccinated controls [98]. Standardization extends beyond single batches. To ensure consistency across manufacturers and over time, international and national reference standards are used to calibrate in-house assays. Additional QC checks include sterility testing to exclude bacterial or fungal contamination, safety testing in sensitive bird models to confirm absence of residual virulence, purity assessments to verify the absence of extraneous agents, and identity confirmation through molecular or serological methods. For novel platforms like vectored or nucleic acid vaccines, developing relevant and robust QC assays, such as tests for genetic stability, expression levels of inserted genes, or lipid nanoparticle characteristics, presents an ongoing challenge. A robust QC system is non-negotiable, as it is the final safeguard ensuring that the vaccine administered in the field performs identically to the vaccine proven effective in clinical trials.

### Feasibility assessment for low-resource endemic settings

The preceding sections describe platforms spanning a wide range of technological maturity, yet the manuscript repeatedly identifies smallholder producers in low-resource, NDV-endemic regions of sub-Saharan Africa and South Asia as the priority beneficiaries of improved vaccine access. This section brings together, in one place, a systematic assessment of whether and how each platform can realistically be deployed in these settings, addressing regulatory pathways, manufacturing geography, cost-effectiveness, deployment timelines, and the non-vaccine requirements that determine whether any vaccine advance translates into field impact.

**Regulatory pathways.** Conventional live attenuated and inactivated ND vaccines are registered through well-established veterinary regulatory authorities across most endemic countries, with review criteria and dossier requirements that are broadly harmonized with regional bodies such as the African Union Pan-African Veterinary Vaccine Centre (AU-PANVAC) [74]. By contrast, no sub-Saharan African or South Asian regulatory authority has yet defined a specific approval pathway for genotype-matched DNA vaccines, VLP vaccines, or other next-generation platforms; applicants would, at present, need to navigate case-by-case novel-product review, and in many jurisdictions the technical capacity to evaluate nucleic-acid or nanoparticle-based products is still being built. Investment in regulator training, reference standards, and mutual-recognition agreements between national authorities is a prerequisite for timely approval of any next-generation platform in these regions, independent of the underlying vaccine science [38].

**Manufacturing geography and capacity.** Conventional egg-based live and inactivated vaccine production already exists, at varying scale, within several endemic-region countries, providing a practical foundation for local supply. Next-generation platforms are a different proposition: VLP and recombinant vector vaccines currently depend on insect-cell, baculovirus, or mammalian-cell manufacturing infrastructure concentrated in a small number of high-income countries, and DNA and mRNA vaccines require specialized plasmid fermentation, lipid-nanoparticle formulation, or electroporation-delivery capacity that is similarly not yet present in most endemic regions [85]. Establishing any of these capabilities locally would require multi-year technology-transfer partnerships and capital investment substantially larger than that needed to expand conventional vaccine output, and facility costs and scale-up timelines for such transfers have not, to our knowledge, been published for the NDV context specifically.

**Cost-effectiveness for smallholder producers.** Conventional live and inactivated NDV vaccines remain, on a cost-per-dose basis, affordable relative to typical smallholder flock revenues in most endemic markets, which is a principal reason for their continued dominance in these settings [55, 111, 112]. Next-generation platforms carry substantially higher per-dose costs, driven by specialized delivery systems (DNA), formulation complexity (VLPs, mRNA), or cold-chain and logistics requirements; while precise, independently verified figures expressing this cost as a percentage of smallholder gross revenue are not available in the published literature and would benefit from dedicated health-economic studies, the qualitative direction is consistent across platforms. In the near term, these platforms are therefore more likely to require donor support, public-sector procurement, or cross-subsidization from higher-value commercial poultry segments than to be adopted through unsubsidized smallholder demand.

**Deployment timeline synthesis.** Table 1 summarizes a realistic, platform-by-platform deployment horizon for low-resource endemic settings, synthesizing the timeline considerations discussed throughout Sections “Conventional NDV vaccine platforms”, “Advanced and emerging NDV vaccine technologies”, and “Regulatory and manufacturing considerations”.

**Non-vaccine requirements.** None of the platforms discussed in this review can substitute for the surveillance, biosecurity, and veterinary-capacity investments needed to translate improved vaccines into sustained disease control. Real-time genomic surveillance is required to detect antigenic drift and inform genotype-matching decisions; basic biosecurity measures (all-in/all-out management, controlled market access, restricted live-bird movement) reduce transmission pressure regardless of vaccine platform; adequately trained veterinary and para-veterinary personnel are needed for correct vaccine handling, administration, and post-vaccination monitoring; and, where cold-chain infrastructure is unreliable, thermostable formulation or alternative stabilization approaches (Section “Field application and real-world performance”) should be prioritized alongside, rather than instead of, vaccine platform innovation.

**Table 1:** Comparative summary of NDV vaccine platforms for low-resource endemic settings

Platform	Immunogenicity profile	Route	Cold-chain need	MDA resistance	DIVA compatibility	Estimated cost/dose	Regulatory status (endemic regions)	Realistic deployment timeline
Conventional live attenuated (lentogenic/mesogenic)	Strong humoral + mucosal	Ocular/nasal, spray, drinking water, in ovo	Required (thermostable strains, like I-2, partially mitigate)	Susceptible to interference	Not DIVA-compatible	Low (~ US$0.01–0.05)	Widely registered and in routine use	Now (already deployed)
Inactivated (oil-adjuvanted)	Strong humoral, limited mucosal	Parenteral injection	Required	Partially resistant	Not DIVA-compatible	Low–moderate	Widely registered	Now (already deployed)
Genotype-matched (reverse genetics)	Strong, better antigenic match	Same as conventional platforms	Required	Similar to parent platform	Depends on backbone	Low–moderate	Not yet approved in most endemic countries; builds on existing pathways	3–7 years
Thermostable formulations	Comparable to parent vaccine	Same as conventional platforms	Reduced/eliminated	Similar to parent platform	Depends on backbone	Low, modest formulation premium	Achievable via existing regulatory pathways for reformulated products	2–4 years
HVT-vectored (rHVT-NDV)	Strong, long-lasting	In ovo or subcutaneous, day-old	Required (though vector itself is stable)	Resistant (vector-based)	DIVA-compatible in principle	Moderate–high	Approved in some markets; limited registration in endemic low-resource regions	Already available; wider access 2–5 years
VLP-based	Strong (experimental)	Parenteral (typical)	Required	Not yet established	Potentially DIVA-compatible	High	No commercial approval anywhere	8–15 + years
DNA vaccines	Moderate–strong with electroporation	Intramuscular or in ovo, electroporation/jet injection	Reduced (thermostable)	Resistant (in principle)	Potentially DIVA-compatible	High (delivery-device dependent)	Preclinical/early experimental; no defined pathway in endemic regions	5–10 + years
mRNA vaccines	Strong (preclinical)	Parenteral (LNP-formulated)	Currently required (cold chain for LNP)	Not yet established	Potentially DIVA-compatible	High	Preclinical; no defined pathway in endemic regions	8–15 + years
AI/ML-informed antigen design	Not yet validated in vivo for NDV	Platform-dependent	Platform-dependent	Platform-dependent	Platform-dependent	Not established	No defined regulatory pathway; concept stage	10–20 + years

MDA: maternally derived antibody; DIVA: differentiating infected from vaccinated animals; LNP: lipid nanoparticle. Cost bands and timelines are indicative syntheses based on the platform-specific evidence discussed in Sections “Conventional NDV vaccine platforms”, “Advanced and emerging NDV vaccine technologies”, and “Regulatory and manufacturing considerations”, and should be refined with dedicated health-economic and regulatory-mapping studies specific to target countries

## Future directions and technological opportunities

### Precision vaccinology approaches

The future of NDV vaccinology lies at the convergence of molecular biology, computational science, and systems-level immunology. Emerging technologies are poised to address the persistent challenges of antigenic diversity, incomplete cross-protection, and logistical constraints, ushering in an era of precision-designed, highly effective, and globally accessible vaccines. Moving beyond the empirical selection of vaccine strains, precision vaccinology applies molecular-level understanding of viral genetics and host immunology to design vaccines that are optimally matched to the specific threat. A primary application is the creation of genotype-matched vaccines tailored to the dominant circulating strains in a given region. Using reverse genetics, the antigenically relevant F and HN genes from a contemporary virulent genotype can be inserted into a proven, safe vaccine backbone. This chimeric approach delivers a vaccine that combines the safety and robust replication of an attenuated strain with the precise antigenic profile of the field virus, potentially narrowing the cross-protection gap [124, 134]. genotype matching, precision approaches can also rationally engineer vaccines to skew the immune response toward a desired phenotype, for instance, enhancing mucosal IgA production or promoting a Th1-biased cellular response, by incorporating specific molecular adjuvants or targeting immunomodulatory genes. This level of precision promises to transform vaccine development from a reactive process to a predictive and tailored discipline.

### Next-generation recombinant platforms

While NDV and HVT have proven to be successful viral vectors, the repertoire is expanding to include novel platforms with unique advantages. Adenovirus-vectored vaccines, for example, offer high levels of foreign gene expression, intrinsic immunogenicity, and established safety profiles in humans and animals, making them attractive candidates for poultry vaccines [44, 66]. Similarly, attenuated bacterial vectors such as Salmonella spp. present a compelling opportunity for oral vaccination. Engineered Salmonella strains can deliver NDV antigens directly to the gut-associated lymphoid tissue (GALT), stimulating robust mucosal immunity through a simple, inexpensive oral dose, a transformative prospect for village poultry systems [37]. The ultimate goal of platform innovation is the development of multivalent “pan-poultry” vaccines. A single recombinant vector, administered once, could be engineered to express protective immunogens from three or four major avian pathogens simultaneously, for example, NDV, IBV, IBDV, and aMPV. Such a product would dramatically simplify vaccination schedules, reduce bird handling and stress, lower production costs, and ensure more uniform coverage across integrated operations.

### Systems biology and immunoprofiling

A deeper, more holistic understanding of the host response to NDV vaccination is essential for rational vaccine improvement. Systems biology employs high-throughput “omics” technologies, including transcriptomics, metabolomics, and immunoprofiling (detailed characterization of immune cell populations), to capture the complete biological response to a vaccine. By analyzing these multi-dimensional datasets in vaccinated versus unvaccinated and challenged birds, researchers can identify novel correlates and mechanisms of protection that extend beyond simple antibody titers [57, 116]. This approach can reveal, for example, why one vaccine platform provides superior cross-protection or longer duration of immunity compared to another. It can identify specific gene signatures or metabolic pathways that predict a robust vaccine “take” or a durable memory response. Ultimately, this knowledge enables the rational design of vaccines that deliberately activate the most effective immunological pathways, moving away from empirical trial-and-error to a hypothesis-driven, data-rich development process.

### AI-driven antigen prediction and vaccine optimization

Artificial intelligence (AI) and machine learning (ML) represent a promising long-term research direction for NDV vaccine development, with the potential to extract actionable insights from vast, complex biological datasets that exceed human analytical capacity, although this potential remains largely theoretical at present. One proposed application is predictive antigen design. AI models could, in principle, be trained on global NDV genomic surveillance data to analyze evolutionary patterns, predict future emergent variants, and identify conserved epitopes, short amino acid sequences that remain unchanged across diverse genotypes. It has been proposed that vaccines targeting such conserved regions could induce broader, cross-genotype protection against current and future strains, potentially mitigating the challenge of antigenic diversity [75].

AI and ML also excel at vaccine formulation optimization. Algorithms can screen thousands of potential adjuvant-antigen combinations, delivery parameters, and stabilization conditions in silico, predicting which formulations will yield optimal immunogenicity, stability, and manufacturability. This reduces the need for costly and time-consuming empirical screening. Furthermore, ML models can integrate disparate data types (genomic, structural, immunological, clinical) to generate a holistic model of protective immunity, guiding the design of vaccines with higher probability of field success [85]. The integration of AI-driven analysis with high-throughput experimental validation represents a powerful feedback loop that will accelerate the pace of innovation and enable a proactive, rather than reactive, stance against the evolving threat of Newcastle disease.

Several substantial gaps currently separate this vision from deployable practice. Published examples of AI-predicted NDV epitopes that have gone on to experimental protective validation in vaccinated birds are scarce; where such epitope-prediction work exists for related paramyxoviruses or other pathogens, its translation to NDV-specific, in vivo confirmed protection has not been demonstrated, and this should be regarded as a theoretical rather than proven capability [47]. The premise of a single universal epitope is also difficult to reconcile with the underlying biology. NDV Class II alone comprises more than 21 recognized genotypes with substantial phylogenetic divergence in the F and HN proteins, and the assumption that one conserved target can confer broad protection across all major circulating variants is optimistic relative to current evidence. Realizing predictive antigen design would additionally require real-time, representative global genomic surveillance capable of detecting emerging variants before widespread dissemination; such surveillance infrastructure is largely absent across most NDV-endemic regions and building it would itself require years of sustained investment in sequencing capacity, sample referral networks, and bioinformatics expertise [47]. Even where an AI-optimized design is identified, manufacturing scale-up and regulatory approval impose the same multi-year timelines as other novel platforms, meaning AI-guided design cannot yet deliver genuinely real-time outbreak response. Taken together, AI/ML-informed antigen design is best framed as a long-term research direction on a 10–20-year horizon rather than an imminent solution and claims of near-term impact should be tempered accordingly.

## Conclusion

In conclusion, the landscape of NDV vaccinology has undergone a remarkable transformation, evolving from empirically derived live attenuated and inactivated vaccines to a new generation of sophisticated platforms, including recombinant vector vaccines, VLPs, nucleic acid vaccines, and rationally designed reverse genetics-based candidates. These technological advances, coupled with innovations in mass application such as *in ovo* delivery and the development of thermostable formulations, have significantly enhanced our ability to control ND in diverse poultry production systems. Despite this progress, critical gaps persist. The continuous emergence and global circulation of novel genotypes have exposed the limitations of conventional genotype II-based vaccines, which often fail to provide sterilizing immunity and allow silent viral shedding. The lack of robust, field-deployable DIVA strategies continues to hamper serological surveillance and eradication efforts, while logistical challenges, particularly cold-chain dependence in low-resource settings, remain formidable barriers to equitable vaccine access. Furthermore, an incomplete understanding of the immunological correlates of cross-protection, especially the relative contributions of mucosal IgA and tissue-resident T cells, impedes the rational design of broadly protective vaccines. Looking forward, the future of ND control lies in the convergence of several transformative technologies. Precision vaccinology, enabled by reverse genetics, will facilitate the rapid creation of genotype-matched vaccines tailored to regional threats. Next-generation recombinant platforms, including adenovirus and bacterial vectors, promise practical, single-dose, orally administrable multivalent vaccines capable of protecting against multiple major poultry pathogens simultaneously. Systems biology and immunoprofiling will deepen our understanding of host-vaccine interactions, identify novel correlates of protection and guide rational antigen and adjuvant selection. Finally, the integration of artificial intelligence and machine learning into genomic surveillance and vaccine design will enable the proactive prediction of emerging variants, the identification of conserved universal epitopes, and the optimization of vaccine formulations. The successful integration of these advanced platforms with practical delivery systems, robust biosecurity, and supportive regulatory frameworks will be pivotal in closing the remaining gaps, ultimately moving the global poultry industry toward sustainable ND control and the long-term goal of disease eradication.

## Data Availability

No datasets were generated or analysed during the current study.
